# Modifying the capacity of shallow foundations to resist uplift forces using structural skirts as a promising alternative to using piles

**DOI:** 10.12688/f1000research.175996.1

**Published:** 2026-04-16

**Authors:** Aqeel Jaafar Al-zubaidi, A’amal A.H. Al-Saidi

**Affiliations:** 1 civil, University of Baghdad Department of Civil Engineering, Baghdad, Baghdad Governorate, Iraq

**Keywords:** Shallow footing, loose sand, uplift capacity, skirt footing, displacement rate, and single piles.

## Abstract

**Background:**

Uplift forces are an important parameter that geotechnical engineers are keen to study for their impact on shallow footings to reduce the risks resulting from them by inventing methods and techniques that limit these effects; one of these methods is the use of piles. This research paper examines the behavior of using structural skirts under shallow footing exposed to uplift loading as a promising alternative to using piles foundations.

**Methods:**

A data logger connected to the loading system, a square footing model, two groups of skirts (chamfered and straight corners) with different properties (length to width ratio, inclination angle, and wings), and two types of single piles with different embedment ratios were utilized under 0.5 and 2 mm/min displacement rates. The behavior and performance of an unmodified footing model, which was placed on loose sand with a relative density of 30%, were examined and compared to those of skirted footing and piled footing under uplift forces.

**Results:**

The findings indicated that augmenting L/B to 2 (where L is the footing length and B is the footing width), the inclination angle to 45° of the skirt with wings, and 0.5 mm/min displacement rate substantially enhanced uplift resistance by 9 times more than the single pile in comparison to the unmodified footing and 10.25 times for L/B to 2, 45° inclination angle with wings, and 2 mm/min, displacement rate.

**Conclusions:**

A significant increase in the uplift capacity of soil was observed by improving its properties by adding a skirt to the shallow footing and making modifications to it using wings. The use of a skirt achieved higher uplift capacity than that obtained from using a pile. Skirted footing demonstrates better performance than the single pile.

**Symbols**
L: skirt length
B: footing width
β: alpha the angle of inclination
D: diameter of skirt
RD: relative density
Ø: angle of internal friction

## 1. Introduction

Geotechnical engineering researchers are still searching for new and innovative ways to improve soil properties to be suitable for bearing the facilities built on them and, on the other hand, to be less costly than traditional methods. They used, for example, geogrid reinforcement, grouting, stone columns, etc. However, it is still governed by the cost and possibility of its implementation on the site. ( Al-Mosawe et al., 2010) conducted tests to improve loose sandy soil using a geogrid model. ( Jawad 2011) conducted a study on slope stabilization using stone columns. ( Shakir 2017) conducted laboratory tests on gypsum soil using Cutback Asphalt as a method of soil treatment to improve its properties (collapse potential and bearing capacity). ( Bachay and Al-Saidi, 2022) conducted laboratory tests to explore the optimal number of geogrid reinforcement layers, which was used to improve the soil under ring footings subjected to inclined loads. ( Daibil and Al-Saidi, 2025) investigated the behavior of a soil-anchor system under different loading conditions that contribute to improving soil.

In recent years, the use of structural skirts in the design of shallow foundations has become prominent, instead of using old traditional methods, like piles. Laboratory model studies were conducted by ( Alhalbusi and Al-Saidi 2023) to examine the behavior of shallow and inclined skirted foundations situated on sandy soil with a relative density of 30%, as well as the influence of positive and negative eccentric-inclined loading effects on these foundations. ( Ahmed et al., 2024) investigated the application of a skirt-raft foundation system, modeled with the finite element software PLAXIS 3D, to assess its efficacy in enhancing stability and protection. ( Al Dabi and Albusoda, 2024) performed an experimental study to evaluate the efficacy of loosely skirted circular footings on badly graded sand, contrasting designs with and without horizontal reinforcing layers in the skirts.

( Al-Mosawi et al., 2011) Found that the piled raft foundation combines piles, a raft, and soil to efficiently distribute applied loads. The piles support loads that may equal or exceed the bearing capability of a single pile, facilitating substantial settlement reduction relative to conventional foundations.( Al-Mosawi et al., 2013) executed laboratory model tests and three-dimensional finite element analysis (ABAQUS) to examine the influence of the pile length-to-diameter ratio and the number of piles on the load-bearing capacity and settlement behavior of piled rafts. ( Al-Wakel et al., 2014) conducted an experimental analysis of the displacement reactions of individual piles and pile groups in dry sand under harmonic (cyclic) excitation. A three-dimensional finite element model was created to replicate the experimental configuration and correctly capture dynamic behavior. ( Salih et al., 2020) indicated that the incorporation of piles can markedly enhance performance when shallow foundations do not meet design specifications, particularly in sandy soils.

### 1.1 Skirted footing

Concrete skirted foundations were used for anchoring the subsea production system (SPS) ( Stove et al., 1992). In jacket constructions, steel skirting footings have effectively taken the place of pile foundations ( Tjelta and Haaland, 1993). Suction anchor piles were used in the Nkossa oil field ( Colliat et al., 1996). (Sherif and Roy, 1998) examined the performance of suction caissons subjected to cyclic and static pullout loading in sandy conditions. The study examined the influence of the suction caisson's length and its performance in drained and undrained scenarios. ( Zdravkovic et al., 2001) examined the influence of various parameters on the pull-out capacity of bucket foundations in soft clay. The parameters include load inclination, skirt length, foundation diameter, soil adhesion, and soil anisotropy. The soil was initially considered isotropic soft clay, represented by a variant of the modified Cam clay model. The study by ( Kelly et al., 2004) discovered that if the up-wind leg(s) could resist significant tensile loads, the efficiency of multiple footing structures using suction caisson foundations would be improved. In a variety of offshore applications, such as wind turbines, oil platforms, offshore industrial facilities, and jacket structures, skirted foundations have proven to be an efficient substitute for surface, pier, and pile foundations ( Andersen et al., 2005).

Using the limit analysis theorem, ( Merifield and Sloan, 2006) performed a numerical investigation to estimate the ultimate pullout force of plate anchors in frictional soils for both vertical and horizontal conditions. The investigation showed that the capacity of vertical anchors was significantly impacted by the roughness of the anchor interface.

(Martinez H.A., 2010) offered experimental results from a group of model experiments conducted by using the beam centrifuge apparatus. This study examined the uplift behavior of shallow skirted foundations for offshore structures exposed to overturning or buoyancy loads. The findings were utilized to examine the mobilization of negative excess pore pressures that improve uplift resistance and their dissipation over time. (Meghdad and Mahmud, 2012) found that the uplift performance of skirted foundations with inclined faces was investigated numerically. The data point to a number of broad conclusions. Compared to vertical-faces foundations, the pullout capability of skirted foundations with inclined sides is reached at greater displacements. The bearing capacity rises with the soil's undrained shear strength (Su), especially for foundations with an inclined skirt surface.

A series of centrifuge model experiments were carried out by ( Xiaojun Li et al., 2013) to assess the uplift strength of rectangular-shaped mudmats placed on mildly kaolin clay under overconsolidated conditions. ( Singh et al., 2013) claim that adding a skirt improves the carrying capacity of a foundation, diminishes settlement, and enhances its load-bearing behavior. The carrying capacity of a ring foundation can increase by 11% to 30%. The pullout load on shallow foundations is much higher than their self-weight. Therefore, this research sought to examine the performance of skirt foundations with embedding ratios (L/D) of 0.5, 1.0, 1.5, 2.0, and 2.5 on loose sandy soil under both dry and flooded circumstances. The undrained response of skirted shallow foundations to uplift and compression has been modeled through large deformation finite element analysis, as demonstrated by (Santiram et al., 2014). The pullout capacity of a skirt foundation is influenced by several elements, including the submerged weight of the skirt, skin friction along the skirt side, the soil plugged within the side of the skirt, the soil shear strength at the skirt end edge, and the suction pressure generated from tension forces across the skirt ( Landlin and Chezhiyan, 2017). The latest experimental studies conducted in a centrifuge are detailed by ( Wang et al., 2019) and ( Xie et al., 2020). These studies examine how the geometry of a skirt influences the uplift capacity of skirt foundations.

The geometry, undrained shear strength, and length to diameter ratio significantly affect the uplift capacity of the skirt foundation. ( Kulczykowski, 2020) presented findings from 1g model tests conducted under single gravity on a skirted foundation placed in sand and exposed to a rapid uplift load. The impact of displacement rates between 5 mm/s and 450 mm/s on ultimate capacity, suction pressure within the skirt compartment, and extraction duration was examined. Test results demonstrate that the displacement rate significantly influenced the magnitude of uplift resistance and the suction under the footing cover, while exerting minimal impact on the relationship between stress and foundation displacement. ( Al –Zubaidi and Al-Saidi, 2025) demonstrated that augmenting the length-to-width ratio to 2 and the inclination angles to 45° of the skirt resulted in a substantial enhancement in uplift resistance, yielding increases of 26 times for skirts with straight corners and 19 times for those with chamfered corners, relative to unmodified footings. It is seen that augmenting the (L/B) ratio has a lesser impact than elevating the inclination angles, as evidenced by 6 times with L=2B and an angle of 0°. Skirt footing with straight corners exhibits superior performance compared to chamfered corners.

### 1.2 Uplift Piles

Uplift piles were commonly employed beneath foundations to resist pullout loads induced by wind and seismic loads, as well as in offshore structures. The most straightforward approach to prevent piles from uplifting is to utilize a pile shaft that is adequately long to accommodate the entire uplift force through shaft friction. Nevertheless, when rock lies behind a shallow soil overburden, it may be infeasible to drive the piles sufficiently deep to activate the necessary frictional resistance. In such instances, the shaft resistance must be enhanced by adding dead weight to the pile to counteract the uplift load or by securing it to the rock. Incorporating dead weight to mitigate uplift loading is either impractical or uneconomical. The piles may need to support alternating uplift and compression loads, whereby the additional dead weight would significantly elevate the compressive loading ( Tomlinson and Woodward, 2007). The maximum uplift capacity of bottom-expansion piles significantly exceeds that of traditional straight piles. The extraction pull-out capacity of belled and multi-belled piles constructed in dense sand was investigated by ( Honda et al., 2011), the belled piles' bearing capacity subjected to pull-out forces increases with the bells' number. ( Mohamed and Amr, 2014) indicated that the skin friction of a pile increases linearly to a constant value governed by the pile embedment ratio and the density of sand. They proposed that a substantial ratio of pile diameter to relative density and buried depth was associated with net pull-out capacity.

Soil trapped within the bells acts like an extension of the pile, augmenting its thickness; as a result, the pile can support more weight because its surface area for friction gets bigger, and the area that carries the load also increases ( Farokhi et al., 2014). The experimental tests were conducted by ( Parthipan and Kumar, 2015) on solid, straight, vertical steel piles with a diameter of 0.8 cm and a length of 15 cm. The sand was utilized in two relative densities: medium and highly dense conditions. A single pile embedded in sandy soil was subjected to testing. The findings indicated that the single pile behavior exposed to pull-out force is contingent upon embedment depth, soil characteristics, and the relative density of the soil. ( Emirler et al., 2020) examined the influence of surface roughness of the pile on the pull-out capacity for various gap ratios among piles in sand. They examined variables encompassing the surface of the pile, the length-to-diameter ratio, and the gap ratio among piles. ( Kang and Kang, 2022) conducted experimental work to investigate the behavior of group piles; 3D analyses using the finite element method were executed to elucidate the pile groups’ failure mechanism and the piles’ interaction for varying gap ratios. The finite element simulation outcomes demonstrate strong concordance with the experimental findings, and all parameters substantially influence the piles' pull-out capacity. The soil located between piles acts as a factor, enhancing the apparent influence diameter of the pile. ( Watanabe et al., 2024) introduced a practical assessment technique for ascertaining the uplift capability and the correlation between uplift resistance and upward displacement of belled piles in sand. Experimental tension studies were performed on different angles of the bell, and the findings indicated that uplift strength fluctuates with the bell angle.

( Nariyelil et al., 2025) investigated the pullout capacity of diverse under-reamed pile designs in two types of soils (saturated clay and clayey sand). Single and double under-reamed piles, half-bulb under-reamed piles, and traditional piles were utilized within various soil classifications. Multiple approaches for evaluating failure stresses and secure pullout capacity were examined to determine their dependability for under-reamed piles. The results indicated that single under-reamed piles featuring a 45° bulb angle and double under-reamed piles with a bulb spacing of 1.5 times the bulb diameter demonstrated improved uplift capacity.

Given the lack of research related to shallow skirt foundations subjected to uplift forces in general and possibility and the economic feasibility of replacing piles with structural skirts, it is imperative to conduct research addressing this important and influential aspect of foundation design. This study investigates the behavior of shallow skirt foundations under uplift loads resting on loose sand and compares it with the behavior of a footing supported by a single pile resting on the same soil to determine whether structural skirts are a promising economic alternative to uplift piles.

## 2. Methods

### 2.1 Research Methodology

**Figure 1.**
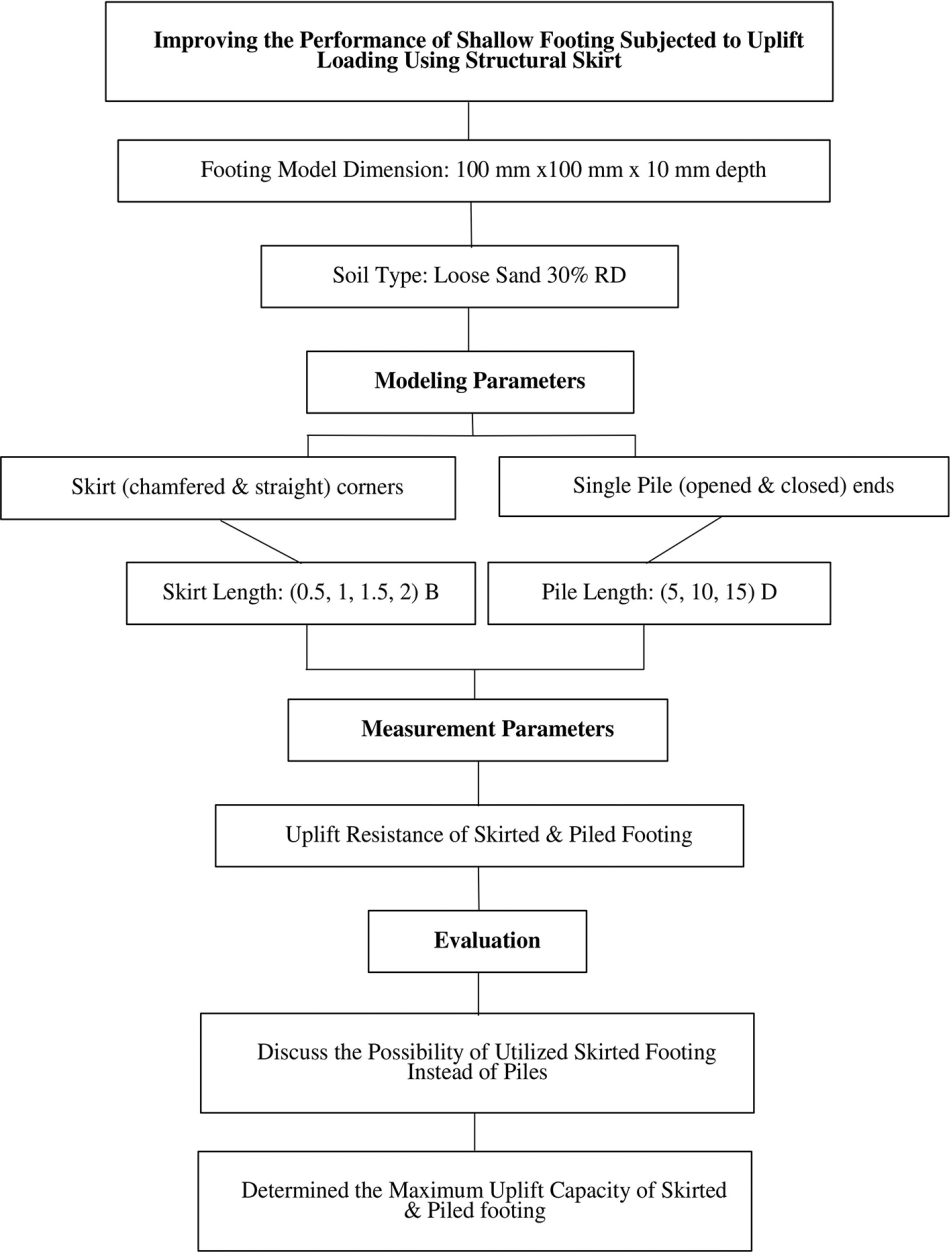
Research Methodology: A flowchart illustrating the research methodology designed to achieve the objectives of this research.

Figure 1 presents a flowchart depicting the research methodology formulated to accomplish the aims of this study. The main aim of this research is to assess the performance of skirted foundations on loose sand under uplift loads and to compare them with single pile foundations to ascertain the viability of utilizing skirts over piles.

### 2.2 Model Box Test

**Figure 2.**
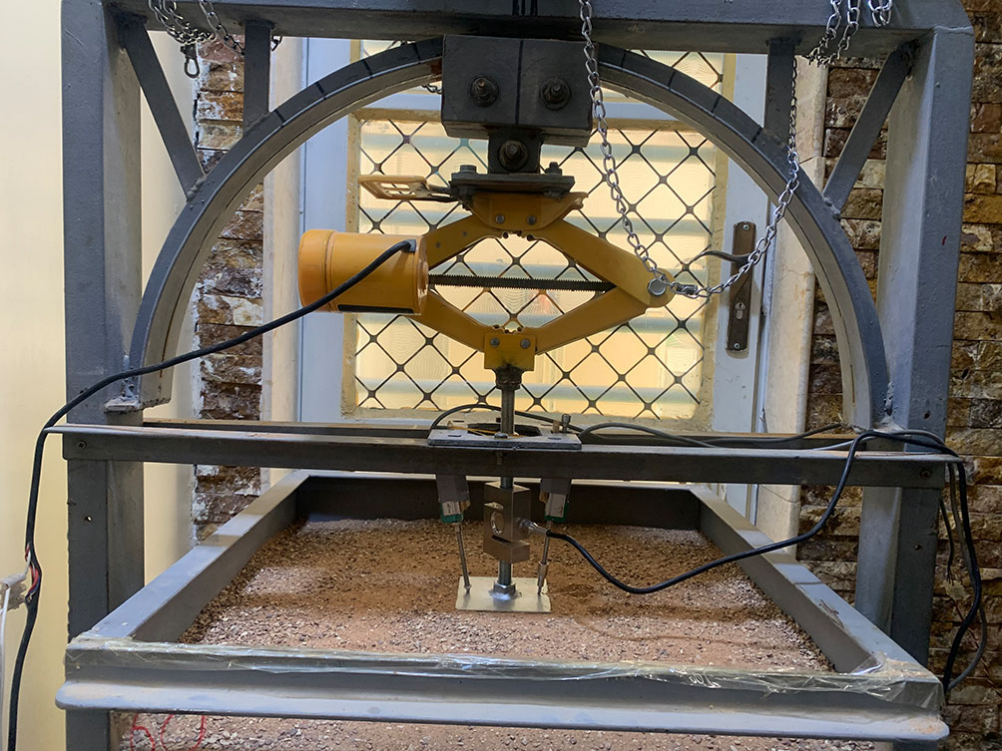
Testing apparatus: The apparatus used for conducting the tests.

The apparatus used for conducting the tests consisted of a steel box as a container for the soil to be tested, with (60 x 60 x 60) cm. The box was manufactured with steel angles as a frame, while steel plates were used to close the sides and base. A glass panel was used at the front to monitor the behavior of the soil and skirted footing during testing. In addition, the loading system consisted of a steel loading were manufactured to support the base of the electric hoist (car jack). The hoist was fixed inverted onto the loading arc to meet the testing requirements and connected to a load cell at the other end, equipped with a transformer to regulate the loading rate. The load cell was connected to a laptop supplied with a data logger to accurately record the readings obtained during the testing. A sand raining technique was used, consisting of a plastic cone with a 25 mm opening diameter suspended by a steel rope to facilitate manual lifting of the cone, while lateral movement was performed manually. A ball valve with the same opening diameter as the cone was located at the end of it to regulate the sand flow rate, which helped achieve the necessary height to obtain the desired relative density. The footing uplift displacement was measured using two linear variable differential transformers (LVDTs). The transformers were connected to a data logger. Figure 2 showed the testing apparatus used.

### 2.3 Model Soil Test

**Figure 3.**
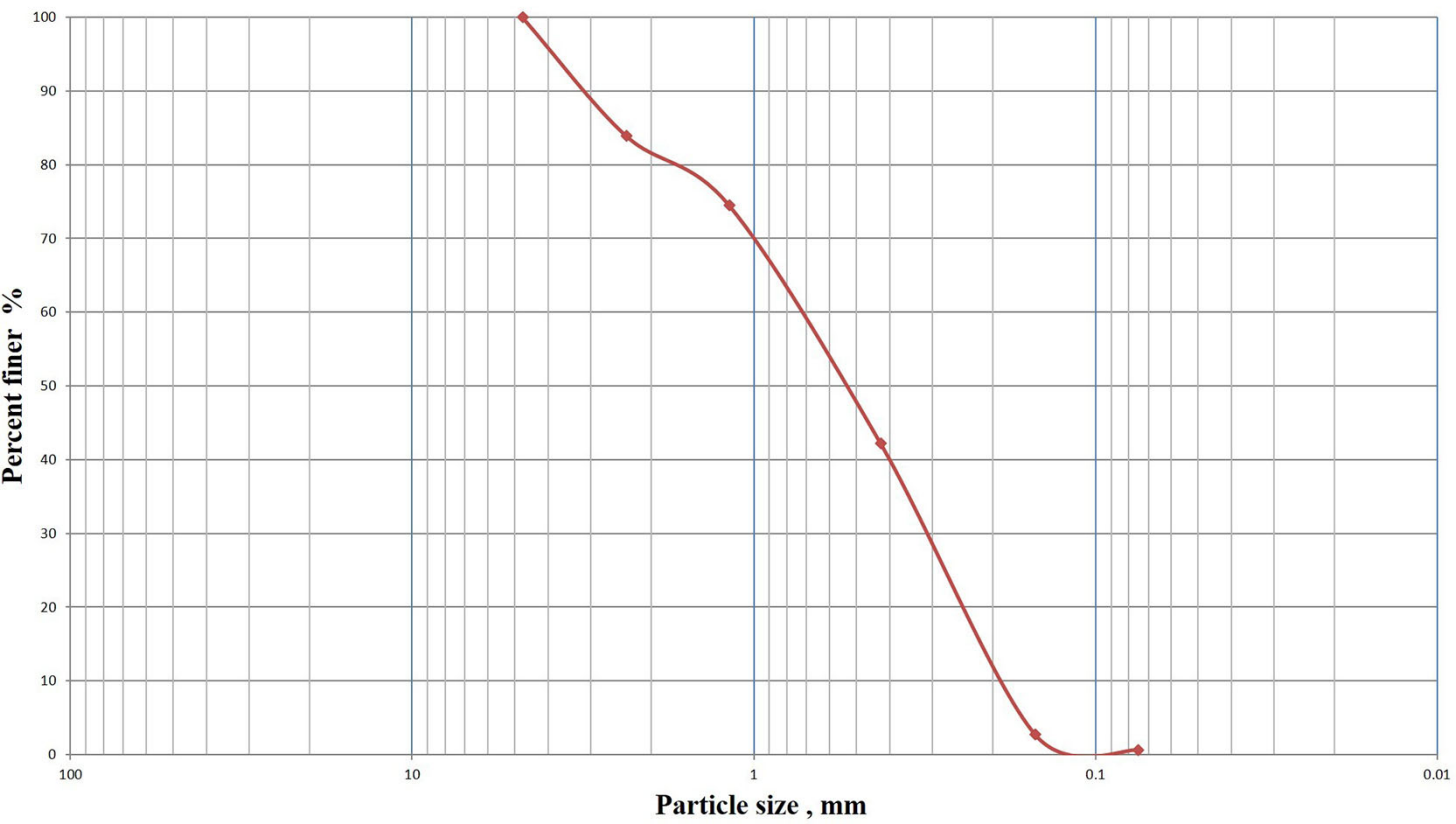
Grain size distribution of the sand: Displays the particle size distribution of the used sand.

This experimental study was conducted utilizing the sands of Karbala. The sand was cleaned, dried, and subsequently sieved through a 4.75 mm sieve before the test. Figure 3 displays the particle size distribution of the used sand. The particle size distribution of the sand utilized was analyzed in accordance with ASTM (D422-63) standard, as depicted in Table 1.

**Table 1.** Physical properties of used sand.

Property index	value	Specifications
Specific gravity (Gs)	2.651	ASTM D-854-92 (ASTM D854–14. 2014)
D10(mm)	0.2	
D30(mm)	0.34	
D50(mm)	0.60	
D60(mm)	0.84	ASTM D-422
Coefficient of uniformity (Cu)	4.188	
Coefficient of curvature (Cc)	0.684	
Maximum dry unit weight (kN/m3)	18.56	
Minimum dry unit weight (kN/m3)	16.09	(ASTM D 4253) & (ASTM D 4254)
Dry unit weight in test (kN/m3) at R.D = 30%	16.76	
Relative density (R.D)%	30	(ASTM D 2049–64)
The angle of interior friction Ø at R.D = 30%	33.7°	ASTM D3080)
Soil classification (USCS)	Poorly graded sand (SP)	Unified soil classification system

To achieve the required relative density for the tests, the raining technique was used. The container was filled with sand in five layers, each layer 100 mm thick, to reach the specified level. This system was developed to achieve this goal. This technique consists of a plastic cone with a 25 mm opening diameter, connected to three steel ropes for balance. This rope acts as a manual lever to facilitate the upward movement of the cone, while the lateral movement was manually executed to distribute the sand evenly across all corners of the container. A ball valve fixed at the exit of the cone to regulate the sand flow rate. Sand flow rate and raining height affect the sand density in the raining technique ( Turner and Kulhawy, 1987). The relative density was maintained at 30% in all experiments to investigate this effect on loose sand. The required level to achieve the required 30% density is 224 mm.

### 2.4 Model skirted footings

**Figure 4.**
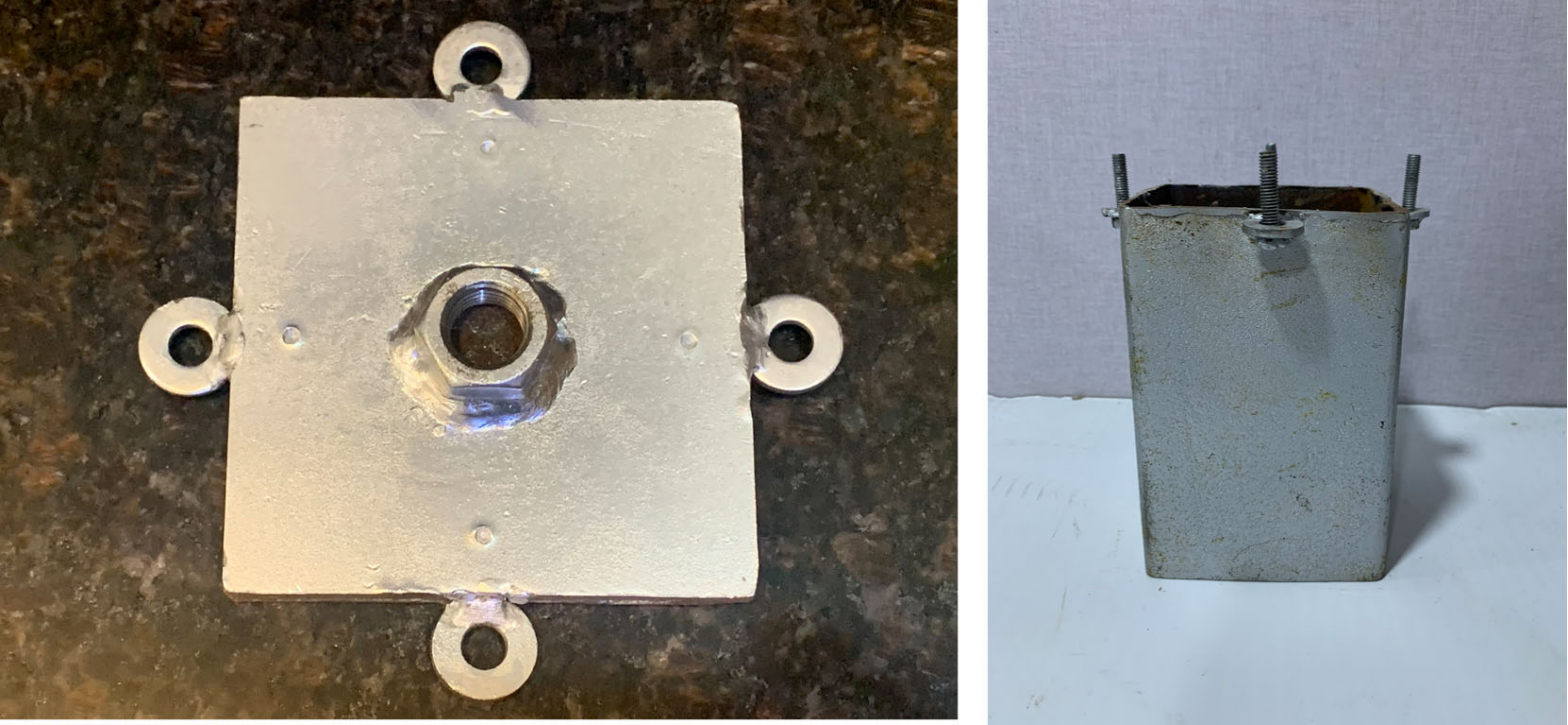
Model Skirt & Footing: A square footing of (10 cm*10 cm) dimensions and a thickness of 1 cm has a nut with a size of 2.7 cm fixed into the surface of the footing model. Four disks and screws are welded on each side of the skirt to connect it with the footing.

**Figure 5.**
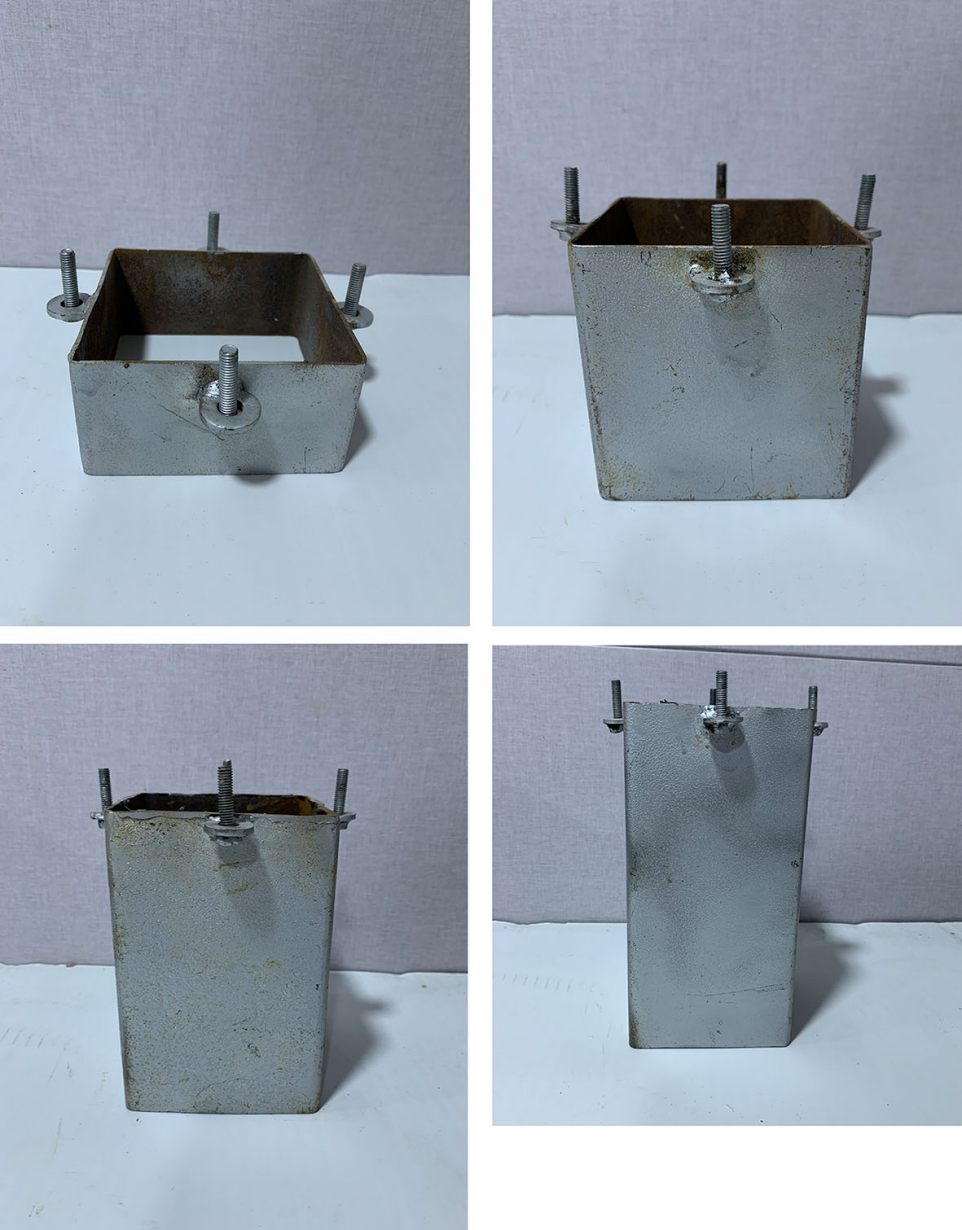
Model Skirt with 0°: The first group of skirts were (0°) inclination angle.

**Figure 6.**
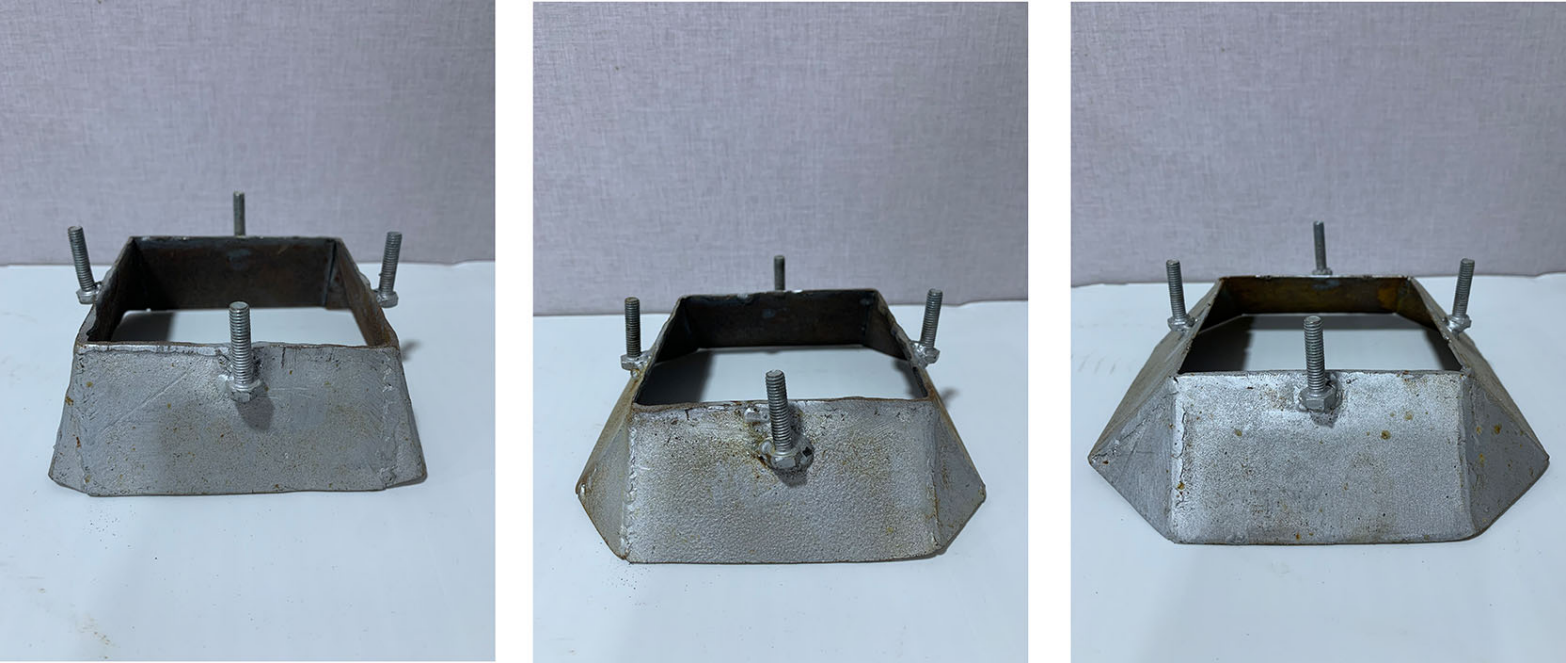
Skirt models with skirt depth 0.5B,chamfer corner & inclination angle (15°, 30°, 45°) respectively.

**Figure 7.**
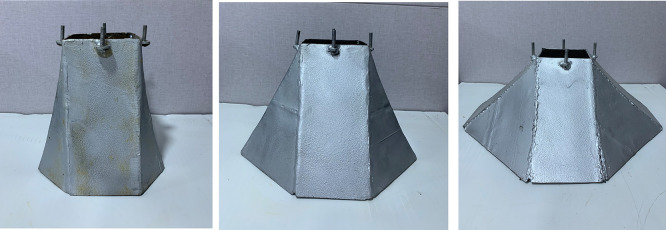
Skirt models with skirt depth 2B, chamfer corner & inclination angle (15°, 30°, 45°) respectively.

**Figure 8.**
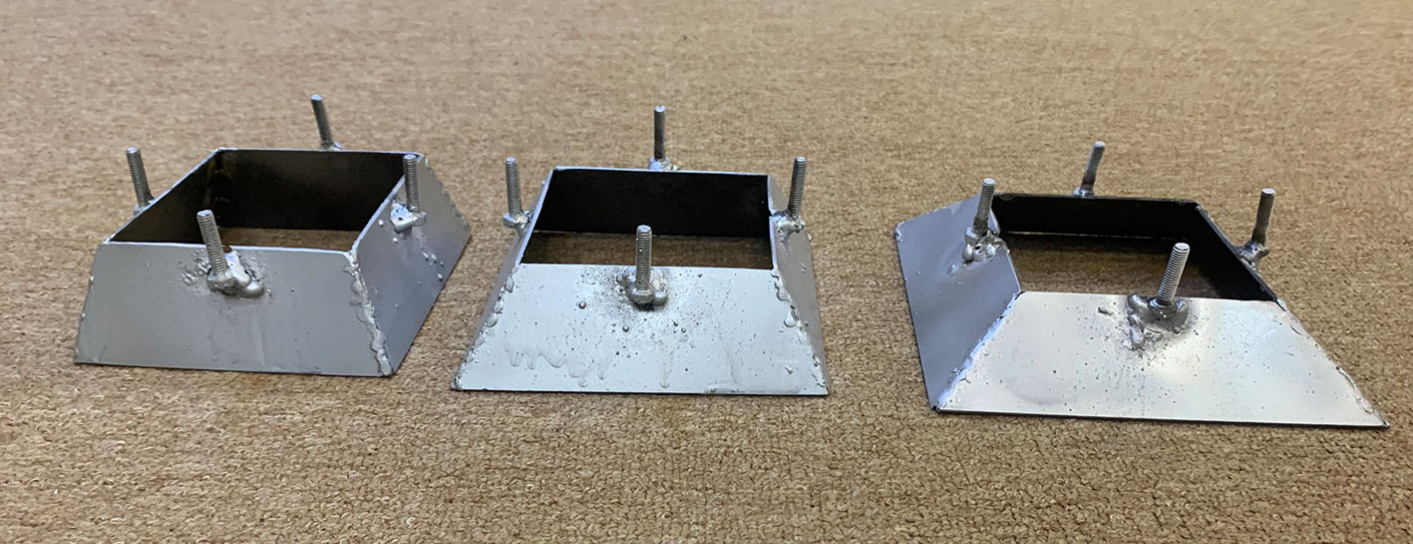
Skirt models with skirt depth 0.5B, straight corner & inclination angle (15°, 30°, 45°) respectively.

**Figure 9.**
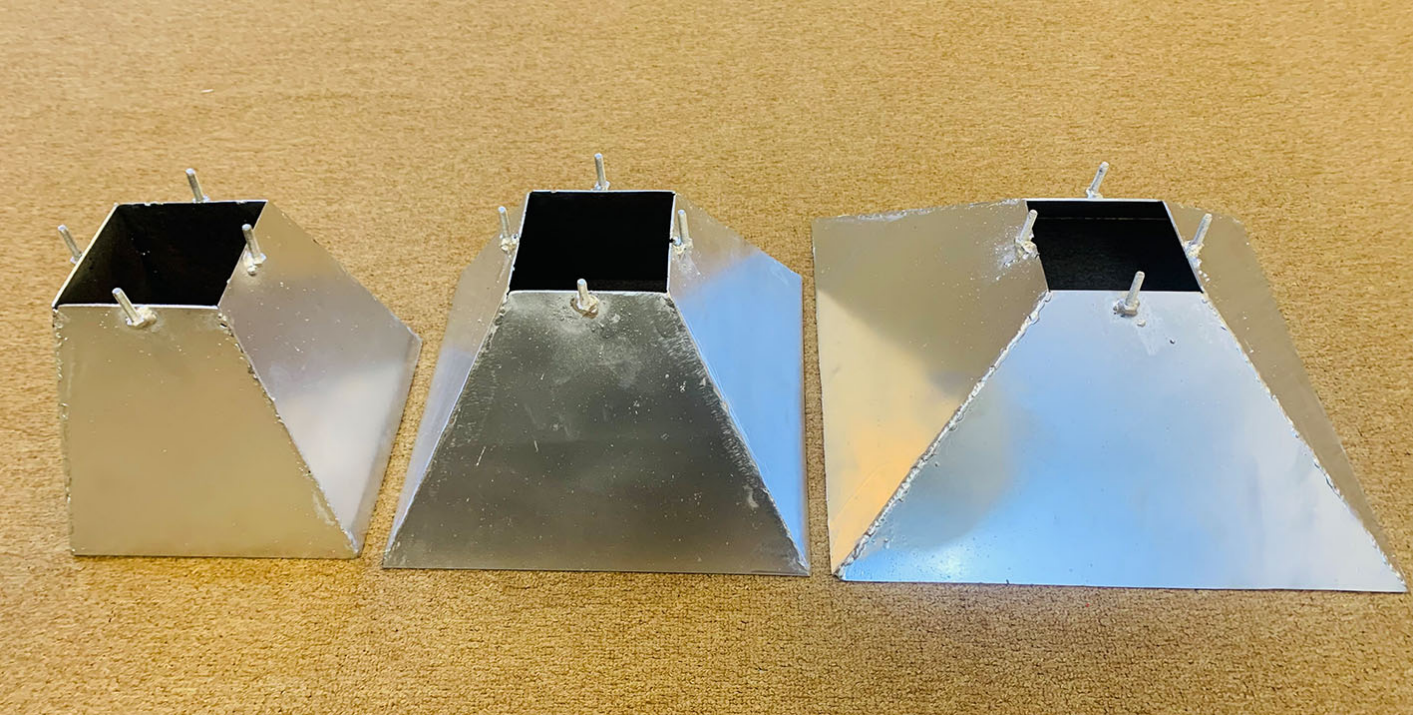
Skirt models with skirt depth 2B, straight corner & inclination angle (15°, 30°, 45°) respectively.

**Figure 10.**
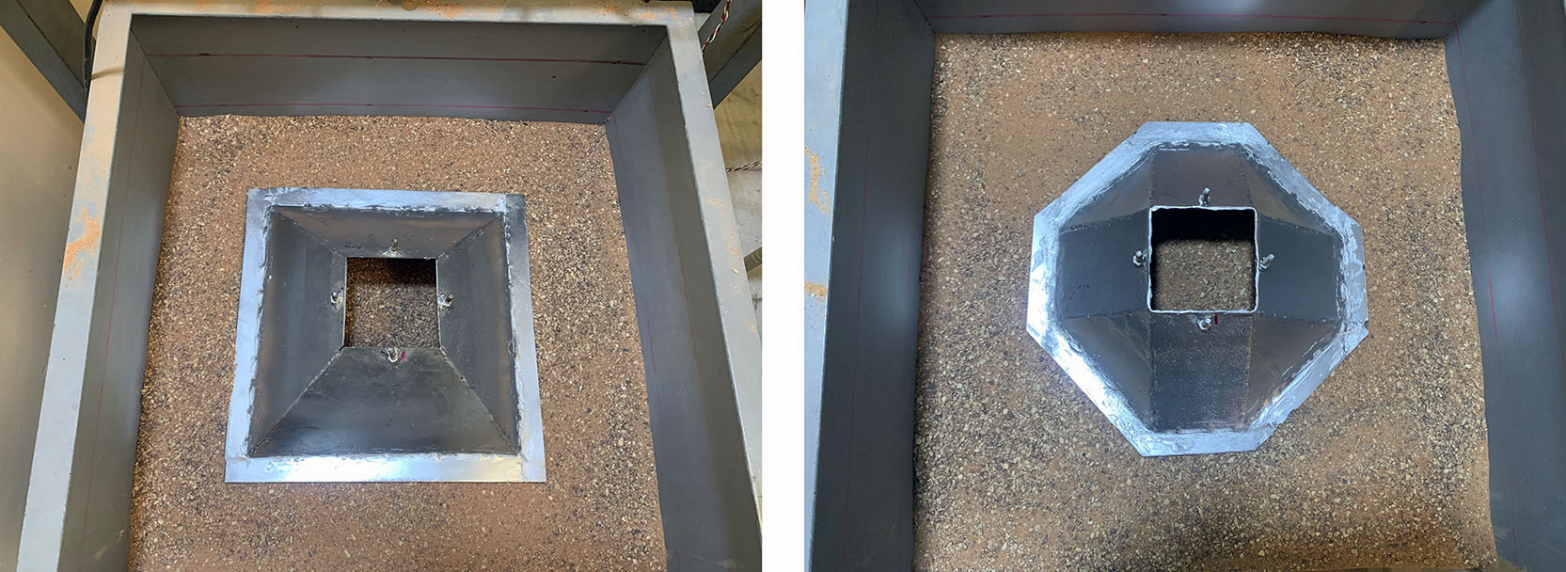
Straight & Chamfer corners skirted model with wings.

**Figure 11.**
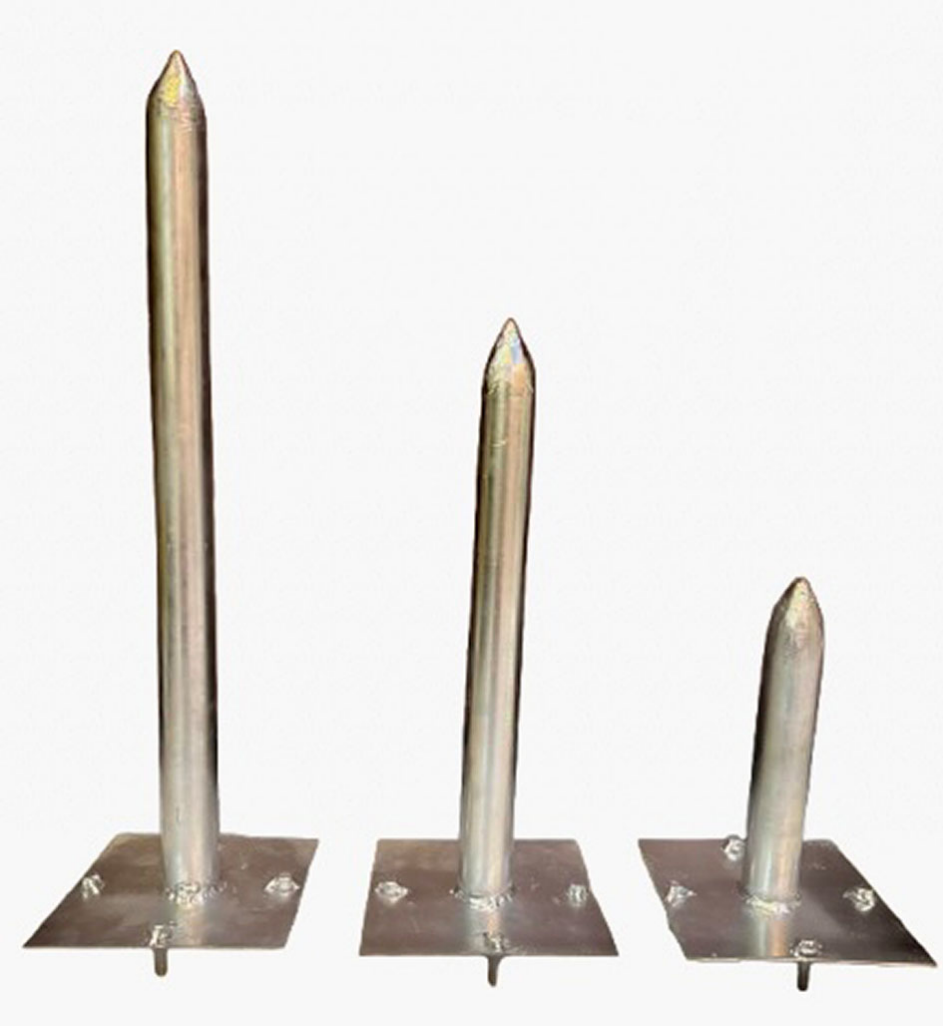
Closed –end pile.

**Figures 12.**
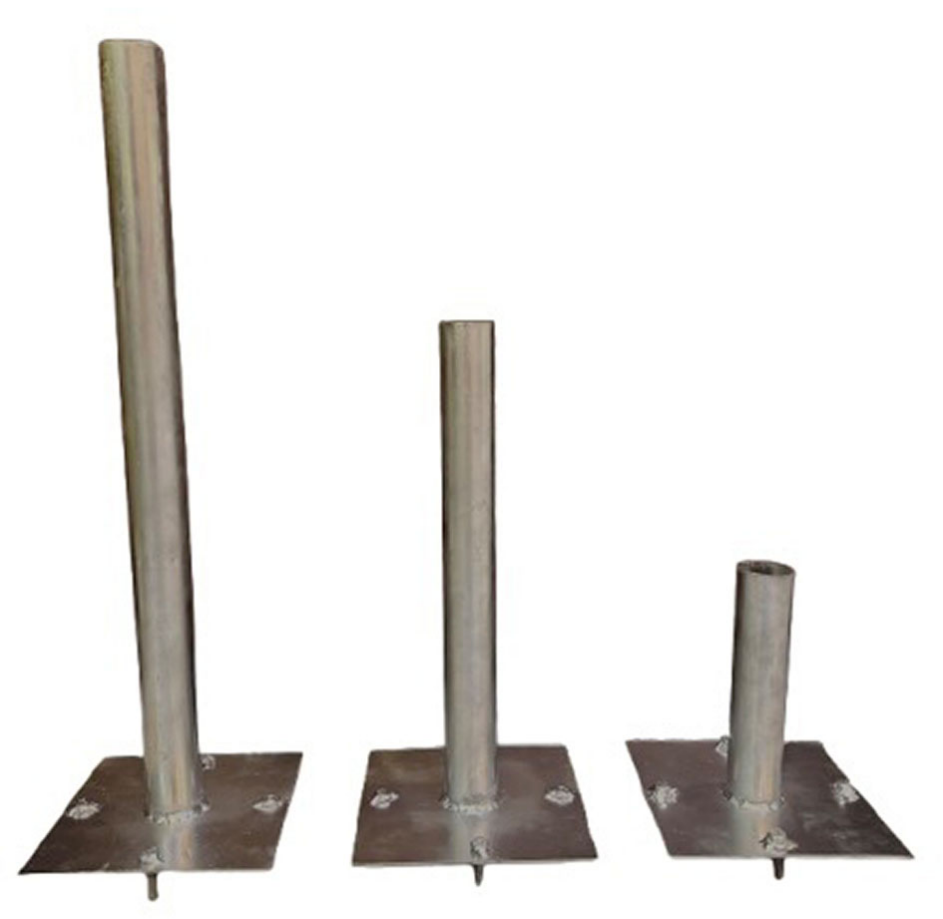
Opened –end pile.

A square footing of (10 cm*10 cm) dimensions and a thickness of 1 cm has a nut with a size of 2.7 cm fixed into the surface of the footing model by a welding technique that was used to connect the footing with the loading arm. Four disks and screws were welded on each side of the skirt to connect it with the footing by corresponding disks that were welded on each side of the footing by nuts, and tiny holes were drilled into the surface of the footing to prevent the (LVDT) from shifting position, as shown in Figure 4. Three groups of steel skirt with 10 cm x 10 cm in dimensions, and a depth of embedment of (0.5, 1, 1.5, and 2) B (where B = width of footing) and 2 mm thick, were used. The first group of skirts were (0°) inclination angle Figure 5. The second group of skirts were chamfered corner (15°, 30° and 45°) inclination angle as illustrated in Figure 6– 7. The third group of skirts were straight corner (15°, 30° and 45°) inclination angles as shown in Figure 8– 9 respectively. The modification has been done on both types of skirts (chamfered and straight) by adding wings to the lower edges of the skirt with 0.25B, Figure 10. Two groups of cylindrical steel piles were used; the first group were closed – conical end, and the conical was 50 mm in length, adding to the total length of the pile. The second group was opened – end. The length to depth ratio (5, 10, and 15) D (where D = diameter of pile), 2 mm wall thickness, and each pile welded with a base plate (14 cm * 14 cm), four screws, and disks 10 mm in size have been welded to each side of the base plate to connect with the footing in the same way that was used with fixing the skirt as illustrated in Figure 11– 12.

## 3. Data analysis

In this research, a comprehensive analysis was conducted of all results obtained from experimental work to test the uplift capacity of shallow footing using different parameters under the same conditions. The analysis used Excel 2013 to plot charts showing the variations in results and make tables showing the percentages of improvement in uplift capacity of sand. The results obtained were compared with those obtained by previous researchers to determine consistency and discrepancy. Using these methods, the study was able to identify the maximum value for optimum uplift capacity of shallow footing and maximum improvement percentage of sandy soil.

## 4. Results and discussions

The primary parameters examined in this research are the skirt geometry, the impact of embedment ratio (L/B), the inclination angles, and adding a wing to the skirt and (L/D) for the single pile on the uplift capacity of shallow footing situated on loose soil. A total of (126) laboratory experiments were carried out, with two displacement rates (0.5 and 2 mm/min), comprising two tests for the unskirted footing, 16 tests for the skirted footing, 48 tests for the chamfered corner, 48 for the straight corner, 6 tests for the footing with closed-end pile, and 6 tests for the footing with open-end pile. Regarding the embedment ratio (L/B), all experiments considered the ratios (0.5, 1, 1.5, and 2) for the skirt and (L/D) (5, 10, and 15) D for a single pile, and the inclination angles 15°, 30°, 45°.

### 4.1 Uplift capacity at 0.5 mm/min displacement rate

**4.1.1 The Effect of Embedment Ratio on uplift capacity at 0.5 mm/min displacement rate**

**Figure 13.**
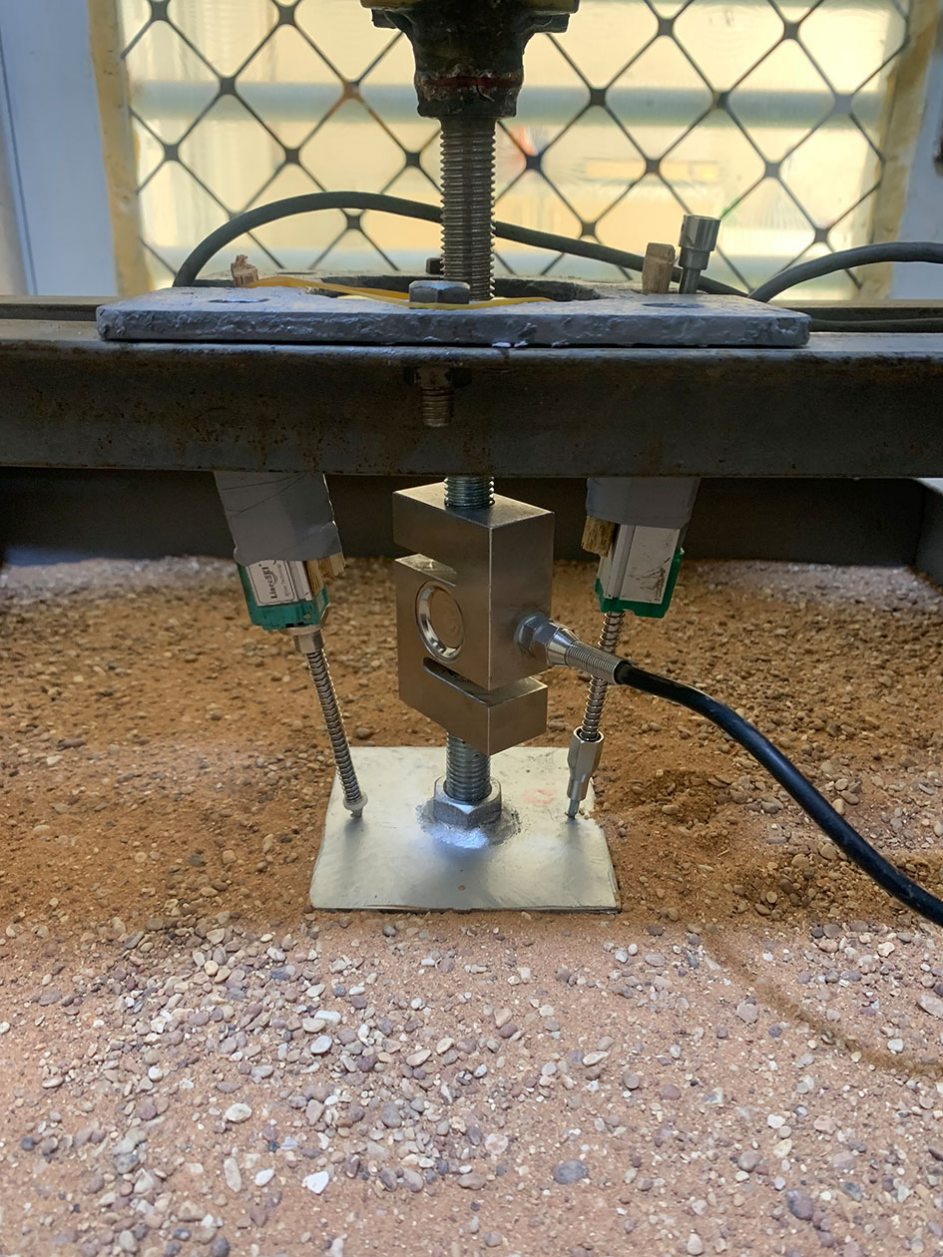
Uplift load applied on unskirted shallow footing.

**Figure 14.**
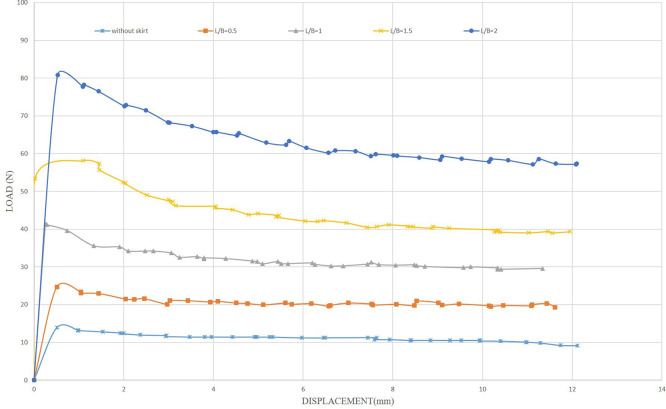
Load-Displacement Relationships of Model Skirts.

The uplift force of the foundation was calculated without modification to be a datum for comparison with the uplift force obtained by making improvements by adding the skirt to the foundation, where the value of the uplift force was 13.88 N, as shown in Figures 13. The initial tests examined the influence of the embedding ratio L/B (where L is the length of the skirt and B is the width of the footing) on the uplift capacity. The uplift capacity of the shallow skirted foundation is proportional to the embedding ratio. By increasing L/B, uplift capacity increased. Figures 14. illustrates the load-displacement relationships for skirt footings.

The load reaches its maximum magnitude at a minimal displacement as the skirted footing model provides resistance to the uplift forces. Following the attainment of the ultimate loading, the loads supported by the skirts progressively diminish as the friction between the soil and the skirt sides decreases linearly. The ultimate uplift observed was 80.76 N for L/B of 2, which is about 5.81 times greater than the skirt footing without modification and 3.27 times greater than the skirt footing with L/B of 0.5, providing maximum resistance of 24.64 N. The optimal depth plays a crucial role in maximizing uplift resistance.

The ultimate load comprises the self-weight of the usual skirt, the weight of the soil confined within the skirt, and the friction between the soil and the skirt's external side. These variables jointly improve anchoring and assist in alleviating the impact of uplift forces on the foundation, especially in wind-prone or seismic regions. The results above comply with the result obtained by ( Landlin and Chezhiyan, 2017) that showed the uplift capacity rises with the ratio L/D of the skirt footing in dry circumstances (where D is the skirt diameter). The maximum uplift capacity recorded was 57.8 N for the model skirt with an L/D ratio of 2.5, approximately five times more than the 11.76 N peak resistance obtained for the L/D ratio of 0.5.

The curve exhibits a zigzag pattern due to the application of a small vertical displacement rate of 0.5 mm/min. The displacement rate is influenced by the resistance of big sand grains (<4.75 mm) when uplift forces are applied. Table 2 shows percent improvement for different L/B ratios of skirts as compared to L/B=0.

**Table 2.** Uplift Capacity Values of Skirted Footings with 100 mm for Different L/B Ratios.

Skirt Length L (cm)	Uplift capacity(N)	Improvement %
0B	13.88	100
0.5B	24.64	177.49
1B	41.39	298.14
1.5B	58.14	418.75
2B	80.76	581.71

**4.1.2 The Effects of inclination angle of model skirt on uplift capacity at 0.5 mm/min displacement rate**

**Figure 15.**
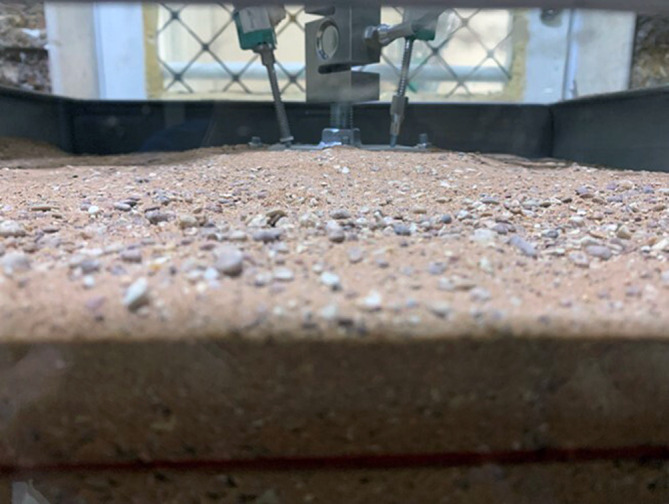
Uplift load applied on chamfered skirted footing with L/B = 1 and β =30°.

The behaviour of resistance to the uplift force in a skirt with an angle of inclination has the same behaviour as that of the skirt when the embedment ratio was increased. Specifically, the maximum force the skirt can achieve occurs within a minimal displacement, after which it gradually diminishes relatively until the residual load is attained. This behaviour includes all L/B of 0.5 and β. In the scenario of a skirt with an increased angle of inclination from (15°, 30°, and 45°), the peak resistance to uplift forces was achieved at minimal displacement, subsequently diminishing with slight displacement. Thereafter, uplift resistance increases again until it attains a maximum at varying displacement values, followed by another decline in uplift resistance. This behaviour can be elucidated by recognizing that the skirt's inclination at a specific angle results in an augmented surface area, thereby enhancing the frictional force with the soil as shown in Figure 15.

**Figure 16.**
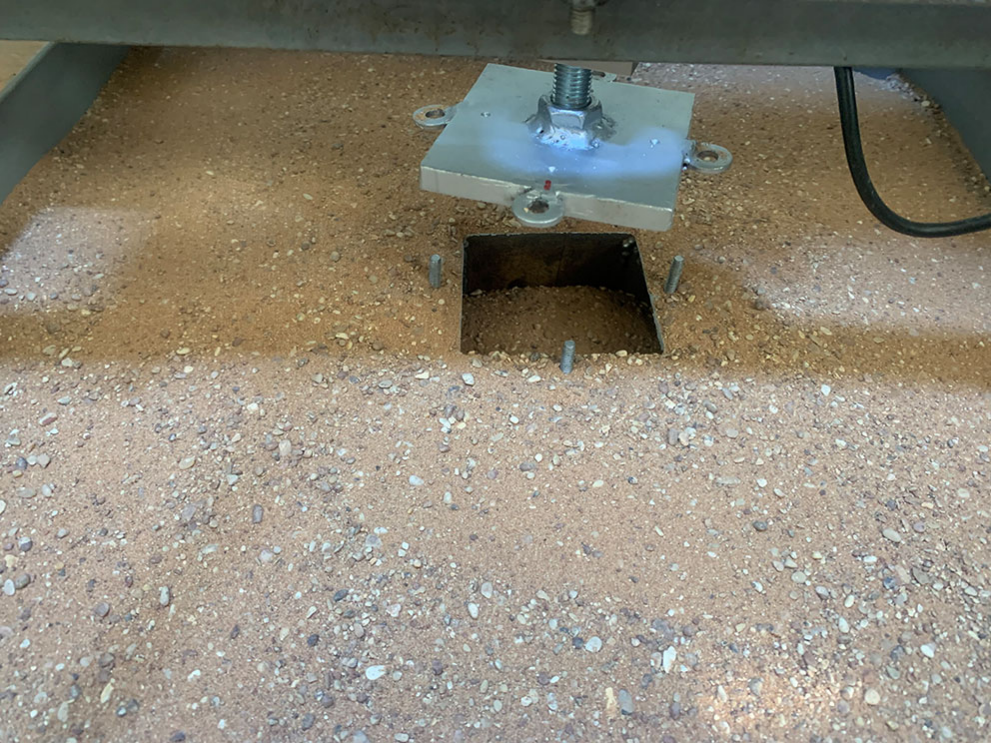
Soil release inside chamfered skirted footing after subjected to uplift load with L/B = 2 and β =30°.

After subjecting the footing to uplift loading, the soil within the skirt released, as shown in Figure 16.

**Figure 17.**
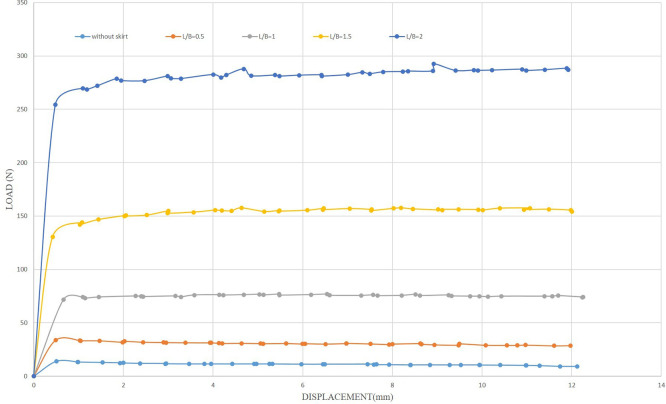
Load-Displacement Relationships of Model Skirts (Chamfered corners) at β = 45°, and 0.5 mm/min Displacement Rate.

**Figure 18.**
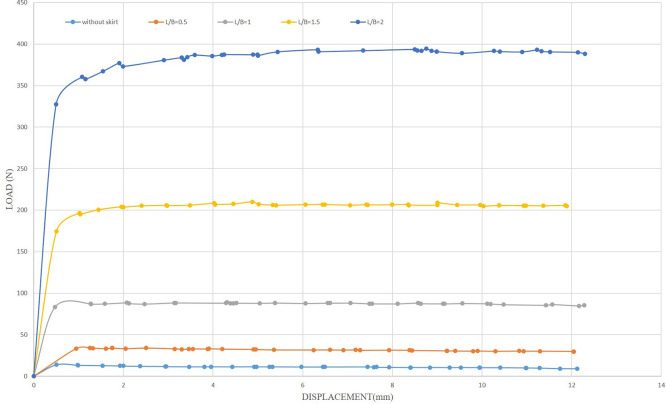
Load-Displacement Relationships of Model Skirts (Straight corners) at β = 45°, and 0.5 mm/min Displacement Rate.

The foundation subjected to vertical uplift loads compels the skirt sides to compress the soil particles above, resulting in their rearrangement and intermingling. As the angle of inclination increases, so does the resistance to uplift. As shown in Figure 17– 18.

**Figure 19.**
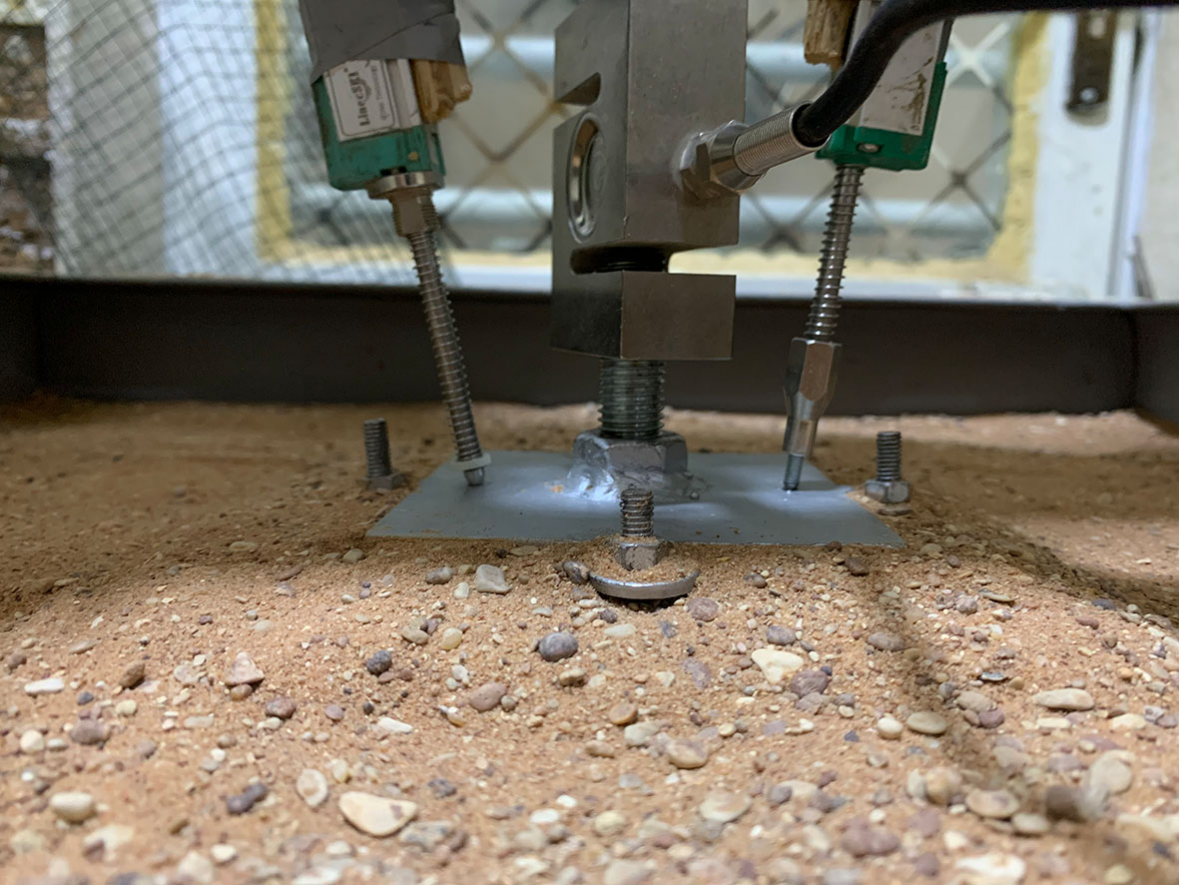
Surface failure of straight -skirted footing under uplift load with L/B = 0.5 and β =30°.

**Figure 20.**
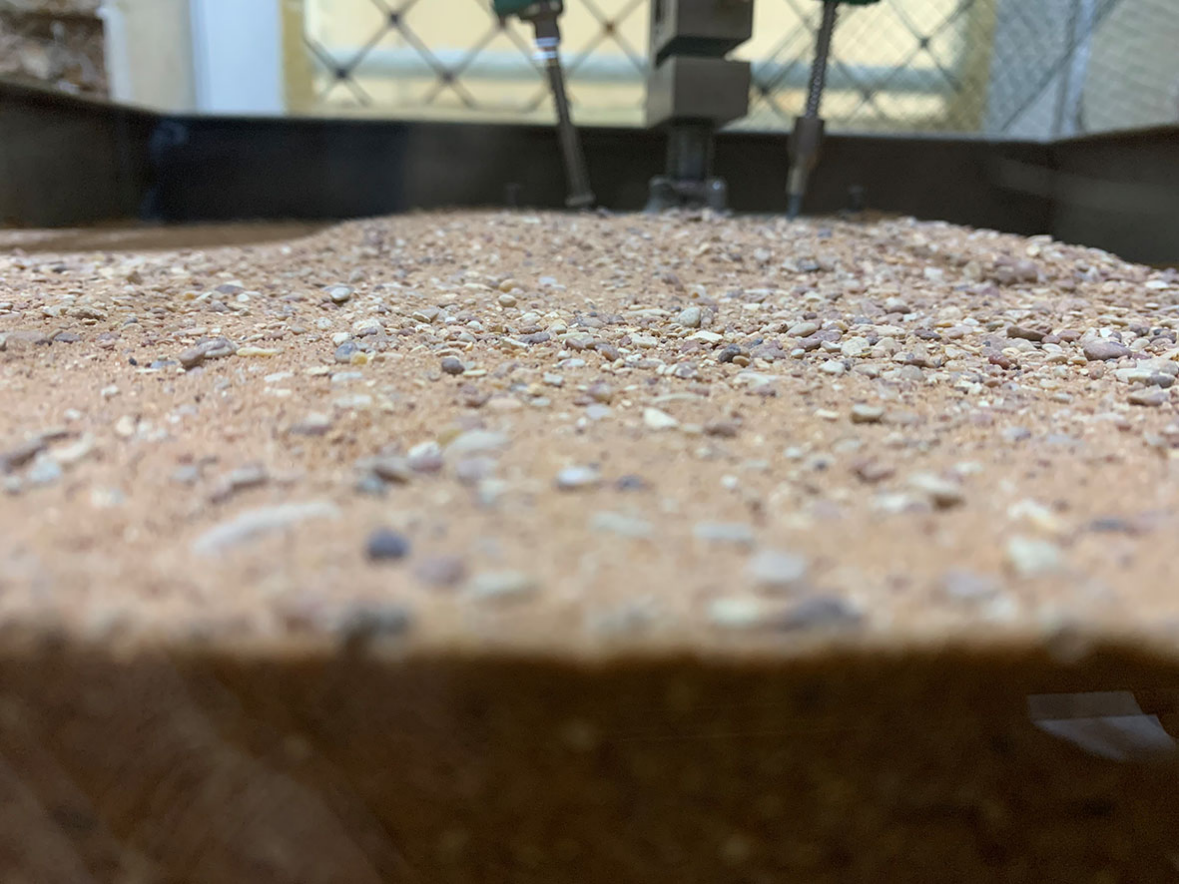
Surface failure of straight -skirted footing under uplift load with L/B = 1.5 and β =45°.

**Figure 21.**
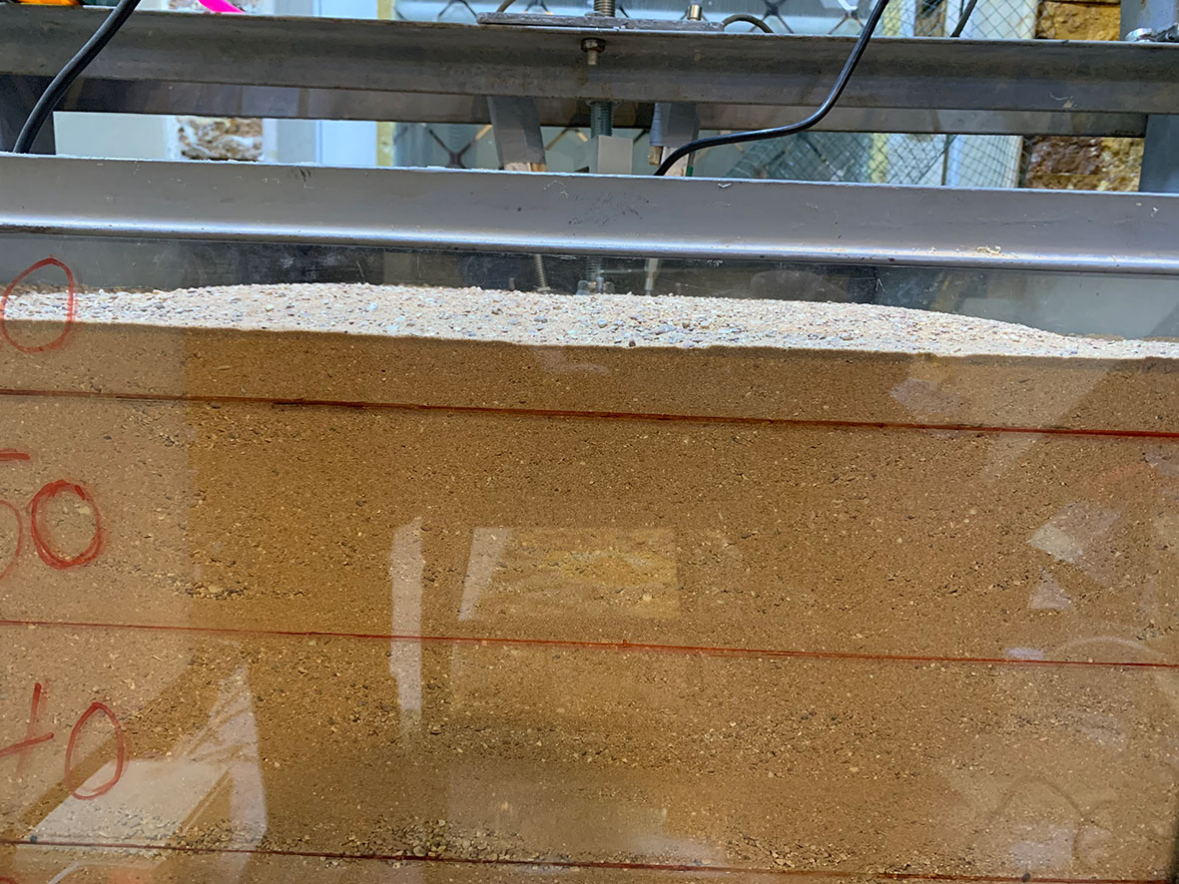
Surface failure of straight -skirted footing under uplift load with L/B = 2 and β =45°.

The ultimate uplift load observed was 33.66 N and 34.14 N for the chamfer and straight corner, respectively, at L/B of 0.5 and inclination angle β = 45°, which is about 2.42 and 2.45 times greater than the skirt footing without modification. In addition, it is 1.36 and 1.38 times greater than the skirt footing with L/B of 0.5, inclination angle β = 0°, and a peak resistance of 24.64 N. The ultimate uplift observed was 269.68 N and 360.47 N for chamfer and straight corner, respectively, at L/B of 2 and inclination angle β = 45°, which is about 19.42 and 25.97 times greater than the skirt footing without modification. Moreover, it is 3.34 and 4.4 times greater than the skirt footing with L/B of 2 and inclination angle β= 0°, having a peak resistance of 80.76 N. Figure 19– 21 showed the surface failure of soil under uplift load.

Table 3 presents the uplift resistance values acquired from the experiments for the chamfered skirt, along with the percentage improvement for each L/B ratio and all inclination angles. Table 4 presents the uplift resistance values acquired from the experiments for the straight skirt, along with the percentage improvement for each L/B ratio and all inclination angles.

**Table 3.** Uplift Capacity Values of Chamfered-Corners Skirted Footings for Different L/B Ratios, B=100 mm, and β, and 0.5 mm/min Displacement Rate.

Skirt Length L (cm)	Skirt Inclination angle (β)	Uplift capacity(N)	Improvement %
0B		13.88	100
0.5B	15°	28.89	208.08
	30°	56.72	408.60
	45°	96.94	698.25
1B	15°	152.45	1098.03
	30°	30.98	223.14
	45°	64.21	462.54
1.5B	15°	125.48	904.03
	30°	210.94	1519.33
	45°	33.66	242.46
2B	15°	73.99	532.90
	30°	143.96	1036.91
	45°	269.68	1942.40

**Table 4.** Uplift Capacity Values of Straight-Corners Skirted Footings for Different L/B Ratios, B=100mm, and β, and 0.5 mm/min Displacement Rate.

Skirt Length L (cm)	Skirt Inclination angle (β)	Uplift capacity(N))	Improvement %
0B		13.88	100
0.5B	15°	28.62	206.16
	30°	54.13	389.91
	45°	114.50	824.67
1B	15°	177.05	1275.19
	30°	30.31	218.31
	45°	80.22	577.75
1.5B	15°	167.16	1204.01
	30°	289.99	2088.68
	45°	34.14	245.90
2B	15°	87.47	629.27
	30°	196.51	1415.38
	45°	360.46	2596.25

The results indicate that uplift resistance does not rise linearly with rising angles of inclination and embedment ratios; instead, the increase is non-linear. The irregular grain distribution of the soil sample, which varies with each experiment, explains this phenomenon. As seen in Figure 1. Shown previously, the percentage of fine material passing through the large-sized sieves (less than 4.75) is less than the usual percentages for well-graded soil, which indicates the presence of high percentages of coarse grains in the soil sample. As known from Table 1. The soil sample is poorly graded, so the grain size was concentrated in disparate regions of the soil container during preparation for testing.

**4.1.3 The Effects of shape of model skirt on uplift capacity at 0.5 mm/min displacement rate**

Tables 3 and 4, as mentioned previously, showed that the influence of skirt shape on uplift capacity is minimal for skirts with chamfered and straight corners at an embedment ratio of 0.5 and an inclination angle of 15°, which is about 28.89 and 28.62 N. However, significant differences in uplift capacity emerge as the embedment ratio and inclination angle increase, with skirts featuring straight corners demonstrating a marked enhancement in resistance to uplift forces, which is about 360.47 N compared to those with chamfered corners, which is about 269.68 N. This behaviour can be clarified by recognizing that the straight-cornered skirt possesses a greater surface area than the chamfered-cornered skirt, hence increasing the area of the skirt subjected to friction with soil particles. Moreover, augmenting the surface area of the skirt increases the quantity of soil confined inside it, raising the overall weight of the foundation subjected to uplift forces. Moreover, augmenting the surface area of the skirt applies pressure to the overlying soil, resulting in the rearrangement and densification of soil, elevating the overall weight of the foundation. The straight-cornered skirt shape demonstrated superior uplift resistance compared to the chamfered-cornered skirt.

There is a great deal of similarity between the shape of the inclination skirted shallow foundations and the belled pile in shape for deep foundations, and it has been observed that their behaviour is largely identical. The use of a bell (which is an enlargement of the base of the pile) acts as an anchor to resist the pullout forces ( Dickin and Leung, 1992; Honda et al., 2011; Gao et al., 2016; Abbas, 2021; and Kang and Kang, 2022).

**4.1.4 The Effects of Adding Wings on Model Skirt on uplift capacity at 0.5 mm/min displacement rate**

**Figure 22.**
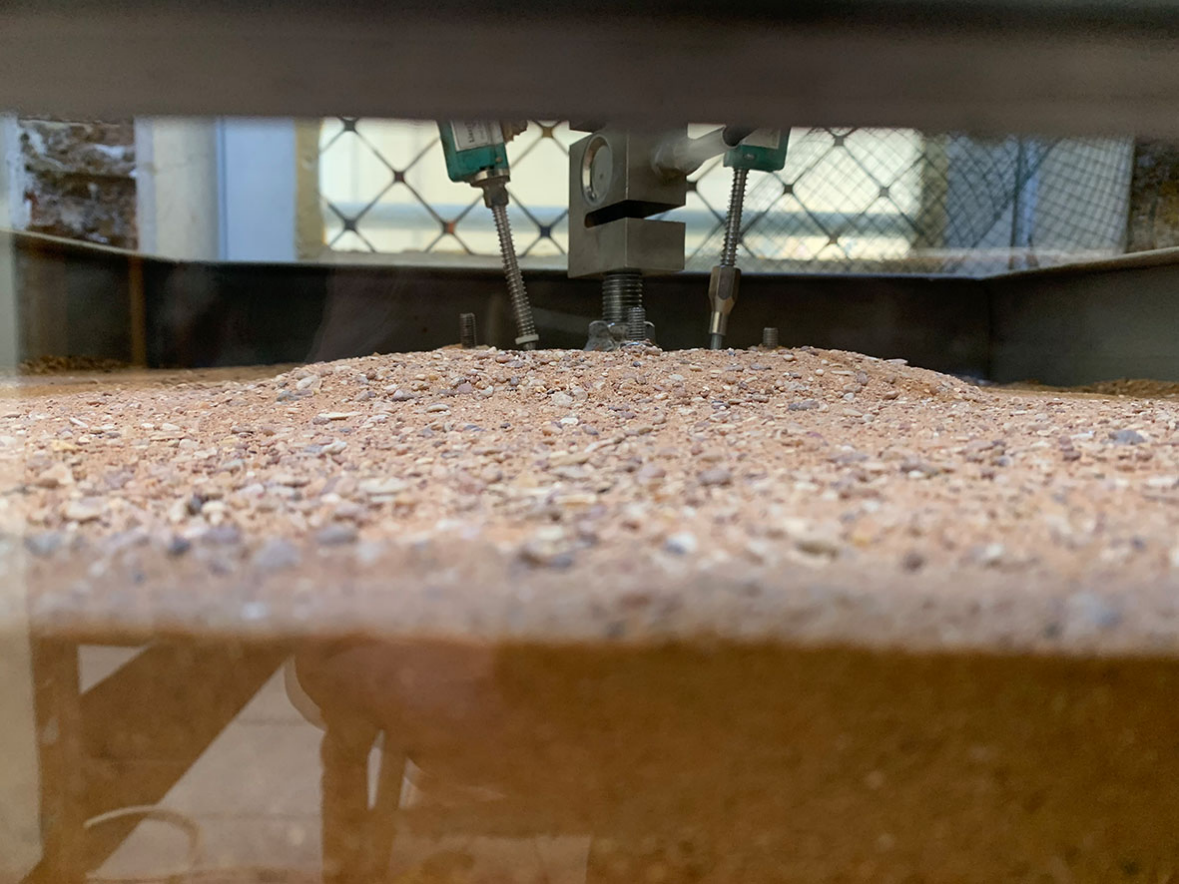
Surface failure of chamfered-skirted footing under uplift load with wings, L/B = 0.5 and β =15°.

A 0.25 B dimension of wings was added to the lower edges of the skirt; as such, the modification gave high values for the uplift resistance compared to the unskirted footing and the skirted footing that was modified by increasing the embedment ratio. Regarding the skirt’s behavior under the influence of the uplift force for both types of skirt by increasing L/B and inclination angles the uplift capacity achieved maximum values. Figure 22. shows the surface failure of chamfered-skirted footing under uplift load with wings.

**Figure 23.**
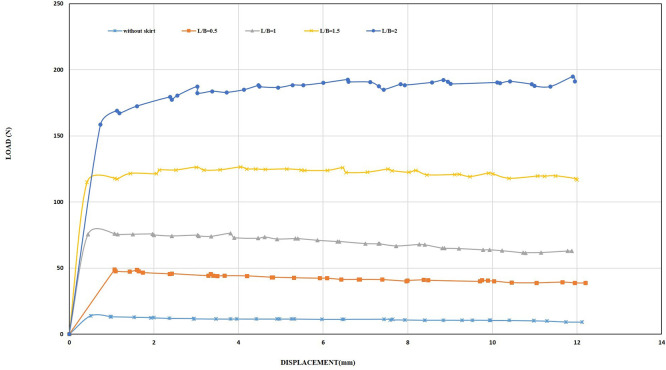
Load-Displacement Relationships of Model Skirts with Wings at β = 0°, and 0.5 mm/min Displacement Rate

Figure 23. shows the load-displacement relationships for skirted footing modified by adding wings with a 0° inclination angle. Results show uplift resistance recorded 48.79 N for the skirt with L/B=0.5, which gives improvement ratios 3.51 times the footing without skirt. The uplift resistance continued to rise until it reached 169.07 N at L/B=2 and remained at an inclination angle of 0°, which gave improvement ratios 12.18 times the footing without a skirt. Table 5. illustrated the results of various L/B and 0° β.

**Table 5.** Uplift Capacity Values of Skirted Footings for Different L/B Ratios, B=100 mm, and β = 0° with Wings, and 0.5 mm/min Displacement Rate.

Skirt Length L (cm)	Uplift capacity(N)	Improvement %
0B	13.88	100
0.5B	48.78	351.39
1B	76.12	548.31
1.5B	117.92	849.35
2B	169.06	1217.72

**Figure 24.**
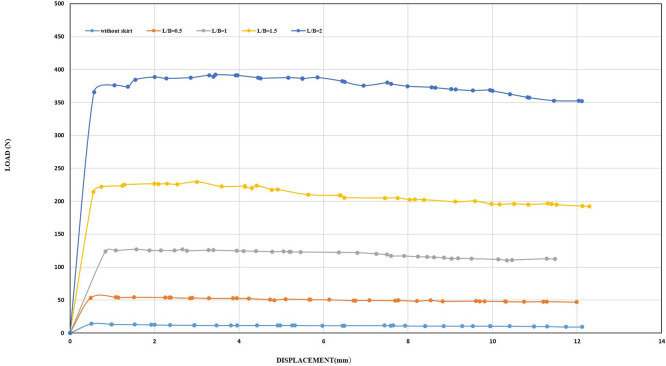
Load-Displacement Relationships of Model Skirts (Chamfered corners) with Wings at β = 45°, and 0.5 mm/min Displacement Rate.

**Figure 25.**
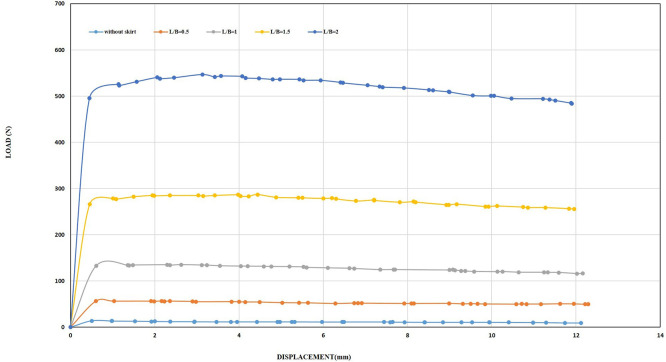
Load-Displacement Relationships of Model Skirts (Straight corners) with Wings at β = 45°, and 0.5 mm/min Displacement Rate.

Increasing the inclination angle of the skirt affecting the uplift resistance of the skirt with wings can be clearly seen from the results compared to the unskirted footing as shown in Figures 24– 25.

The uplift force for the chamfered and straight skirts at 15° inclination angles and an embedding ratio of 2 was 251.80 and 303.82, which are 18 and 22 times greater than the footing without a skirt. With increasing inclination angles up to 45°, the results indicated 376 N for the chamfered skirt and 525.59 N for the straight skirt, which are 27 and 37.86 times, respectively greater than the unmodified footing.

The reason for the increased uplift resistance is that the additional wings will anchor the skirt to the ground, and the inclination of the skirt at various angles serves as an anchor and increases the surface area of the skirt. In addition, the increased embedment ratio increases the frictional force between the soil confined within the skirt and the surrounding soil. Tables 6 and 7 illustrated the results of uplift load of various L/B and β.

**Table 6.** Uplift Capacity Values of Chamfered-Corners Skirted Footings for Different L/B Ratios, B=100 mm, and β with Wings, and 0.5 mm/min Displacement Rate.

Skirt Length L (cm)	Skirt Inclination angle (β)	Uplift capacity(N)	Improvement %
0B		13.88	100
0.5B	15°	51.07	367.83
	30°	93.81	675.71
	45°	171.31	1233.92
1B	15°	251.79	1813.57
	30°	52.67	379.37
	45°	120.94	871.11
1.5B	15°	216.22	1557.38
	30°	356.62	2568.57
	45°	54.26	390.82
2B	15°	127.14	915.79
	30°	226.56	1631.84
	45°	376.00	2708.16

**Table 7.** Uplift Capacity Values of Straight-Corners Skirted Footings for Different L/B Ratios, B=100 mm, and β with Wings, and 0.5 mm/min Displacement Rate.

Skirt Length L (cm)	Skirt Inclination angle (β)	Uplift capacity(N)	Improvement %
0B		13.88	100
0.5B	15°	50.16	361.34
	30°	101.14	728.47
	45°	179.22	1290.87
1B	15°	303.81	2188.23
	30°	54.94	395.73
	45°	125.43	903.45
1.5B	15°	258.35	1860.81
	30°	433.31	3120.95
	45°	56.18	404.66
2B	15°	134.07	965.65
	30°	278.60	2006.67
	45°	525.59	3785.58

Results obtained from the added wings to skirt comply with some researchers regard working mechanism ( Dickin and Leung, 1992); ( Ilamparuthi and Dickin, 2001); ( Honda et al., 2011); ( Mohamed and Amr, 2014); (Diana et al., 2017); ( Abbas, 2021); (Jebur et al., 2020).

**4.1.5 The uplift capacity of single pile at 0.5 mm/min displacement rate**

**Figure 26.**
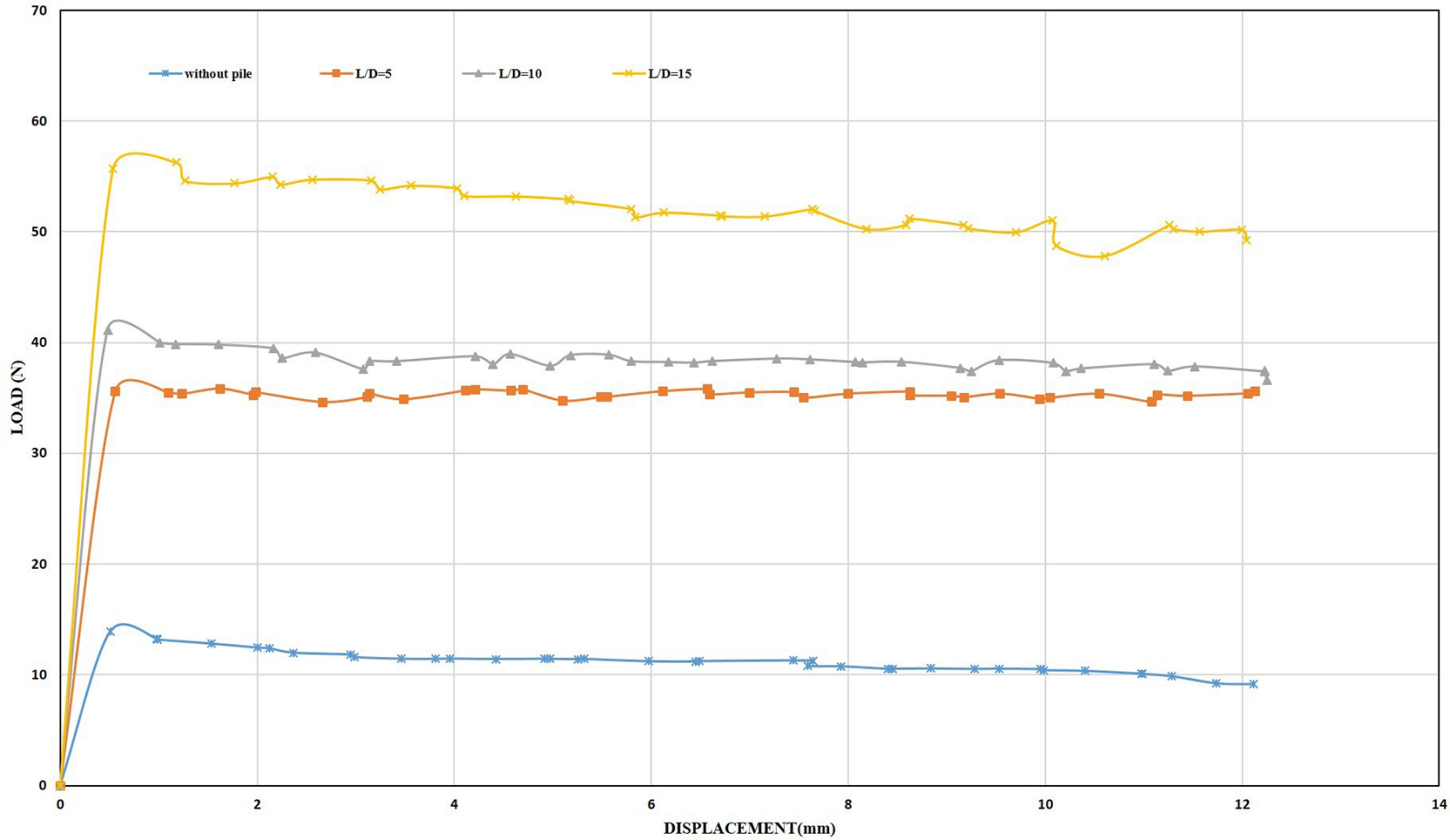
Load-Displacement Relationships of Single closed end pile.

**Figure 27.**
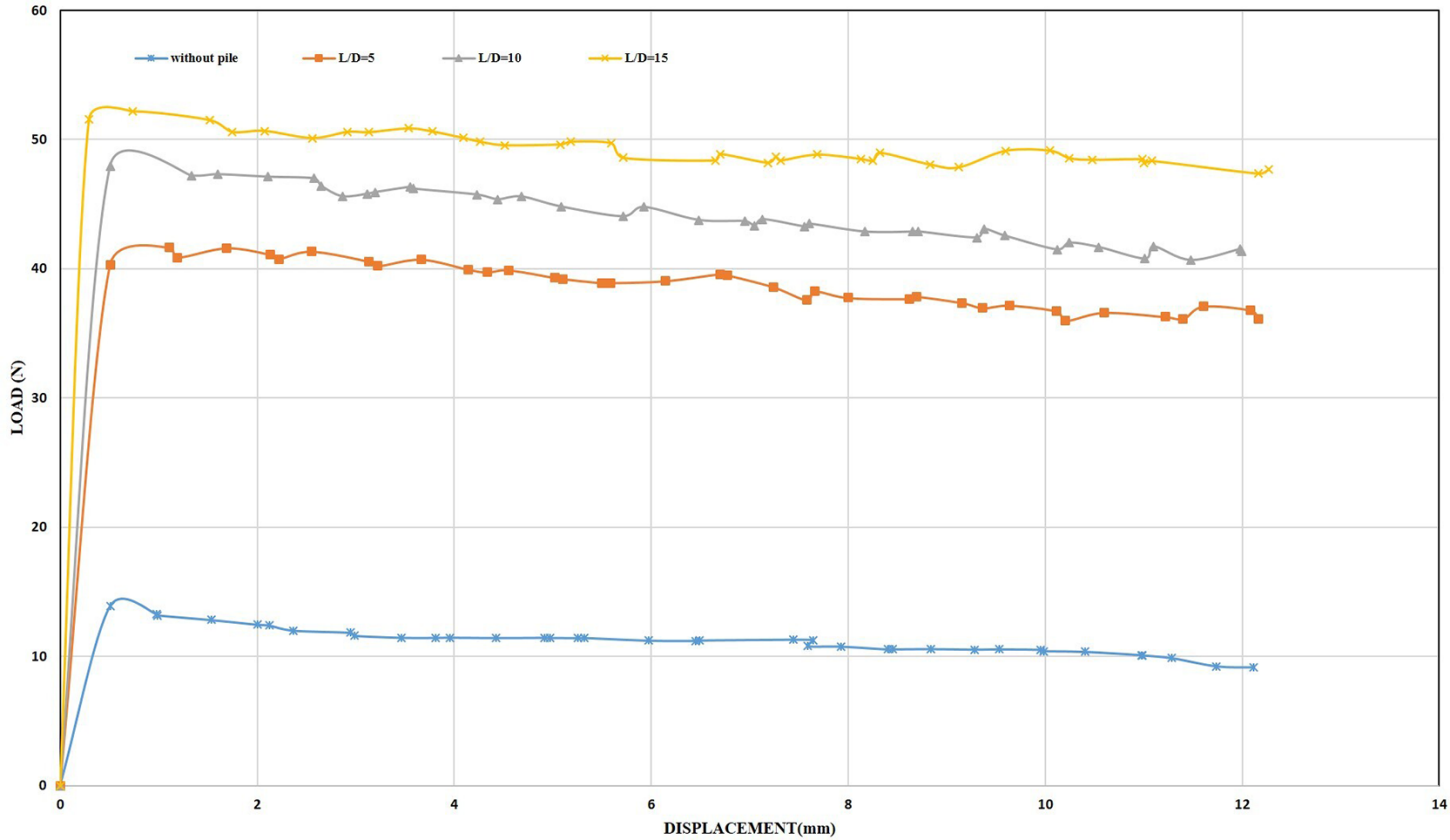
Load-Displacement Relationships of Single opened end pile.

Figure 26– 27 shows the results obtained from laboratory tests at a displacement rate of 0.5 mm/min.

Although the pile was inserted into the soil sample for a distance of 8 cm of the pile length, which is equivalent to (53, 26, and 17)% of the total length of the pile for embedment ratios (5, 10 and 15) D, respectively, which means that the pile gained strength under compression while applying load on it by the loading system. However, the maximum uplift resistance obtained was 56 N for a single closed-end pile at an embedment ratio of 15, which was lower than the results obtained when using a structural skirt at an embedment ratio of 2 and a 0° inclination angle, which equals 80 N, which is 1.42 times more than the uplift capacity of the single pile.

**Figure 28.**
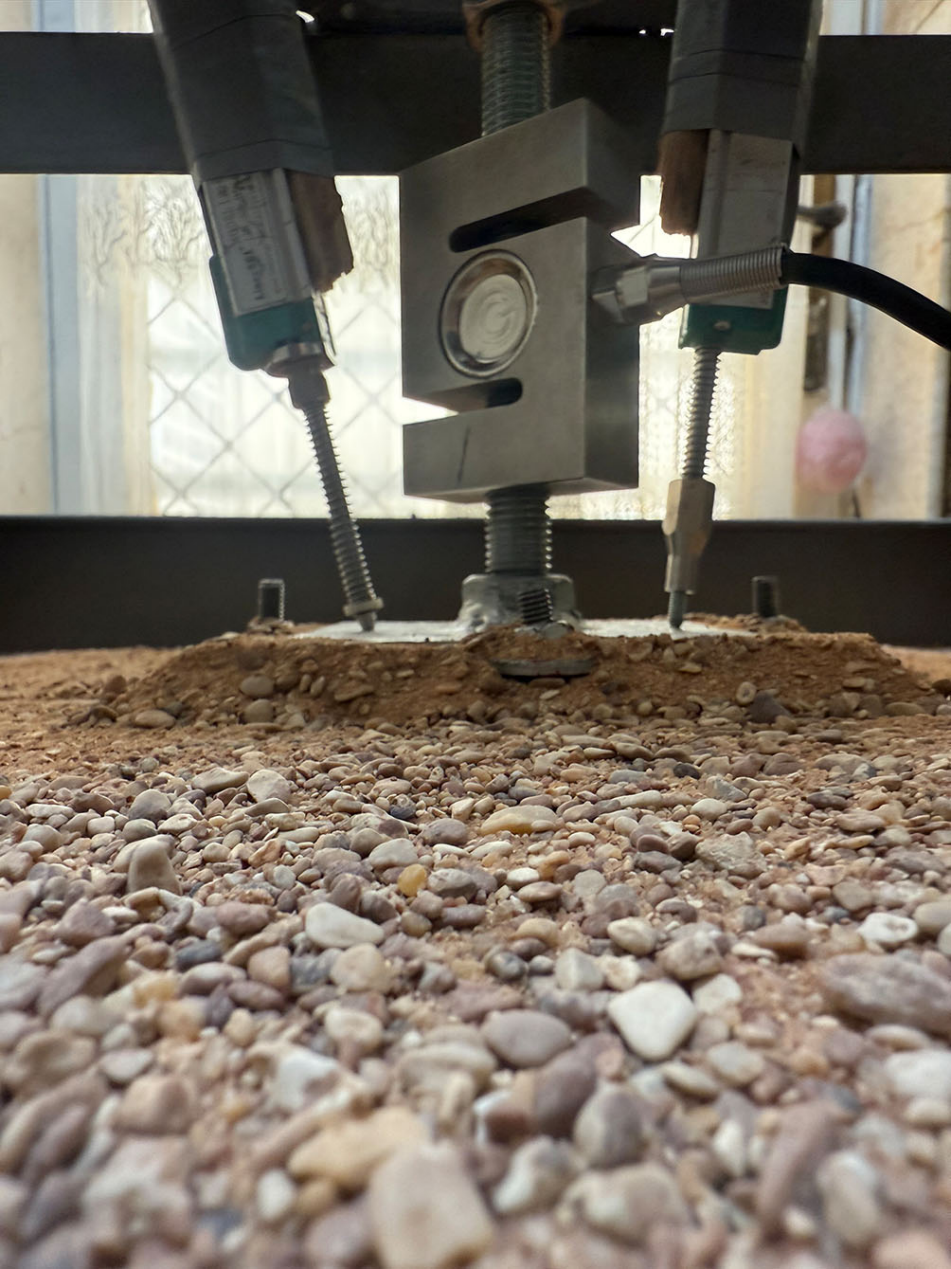
Surface failure of single pile footing under uplift load.

The results obtained from testing a single closed-end pile at L/B ratio of 5 were 35 N, which is lower than the results obtained for the same embedding ratio of the structural skirt, which was 58 N, which was 1.65 times greater than the uplift capacity of the single pile. The maximum uplift resistance obtained when using a skirt was 525 N for a skirt with wings, an embedding ratio of 2, and an inclination angle of 45°, which is 9 times greater than the uplift capacity of a single closed-end pile at 15 L/B ratios. Hence, it has been demonstrated that a structural skirt under shallow foundations subjected to uplift loads is a viable, economical alternative to deep foundations. Figure 28 showed the surface failure of a single pile footing under uplift load.

It is worth noting that the length of skirt for embedding ratio 2 is 20 cm, while the length of the single pile for embedding ratio 15 is 45 cm. Table 8 and 9 shows the test results and the percentage improvement in uplift capacity provided by single steel piles at a displacement rate of 0.5 mm/min. As for the open-ended piles, the results obtained were less than the closed piles for the embedment ratio of 15, but they gave higher results for embedment ratios of 5 and 10. This is due to the difference in the grain distribution of the sand when preparing the sample for examination.

**Table 8.** Uplift Capacity Values of Single Pile Footings for Different L/D Ratios of Closed End Piles, and B = 100 mm, and 0.5 mm/min Displacement Rate.

Pile Length L (cm)	Uplift capacity(N)	Improvement %
Unskirted footing	13.88	100
5D	35.60	256.42
10D	41.14	296.37
15D	56.25	405.15

**Table 9.** Uplift Capacity Values of Single Pile Footings for Different L/D Ratios of Opened End Piles, and B = 100 mm, and 0.5 mm/min Displacement Rate.

Pile Length L (cm)	Uplift capacity(N)	Improvement %
Unskirted footing	13.88	100
5D	41.64	299.94
10D	47.92	345.16
15D	52.17	375.82

### 4.2 The uplift capacity at 2 mm/min displacement rate

As was done in the first group of tests, the load-displacement relationships and the ultimate uplift load of footings with and without skirts were obtained, but with 2 mm/min displacement rate. By applying the same parameters used in the test. The purpose of increasing the displacement rate is to simulate the rapid uplift that the footing may be exposed to. An uplift displacement rate of 2 mm/min was used due to the limited capabilities of the testing device. When the capabilities of the testing device were high, the tests would have been carried out at different displacement rates until the footing was completely extracted from the soil to understand the behavior of the footing under rapid uplift loads more broadly.

Tests were carried out on footing without a skirt as a reference test for comparative analysis. Increasing the uplift displacement rate from 0.5 mm/min to 2 mm/min did not significantly change the general behavior of the footing with and without a skirt, but some changes occurred that were observed through the results obtained from the tests, which are shown with each of the parameters on which the footing was tested.

**4.2.1 The Effect of Embedment Ratio of Skirt on uplift capacity at 2 mm/min Displacement Rate**

**Figure 29.**
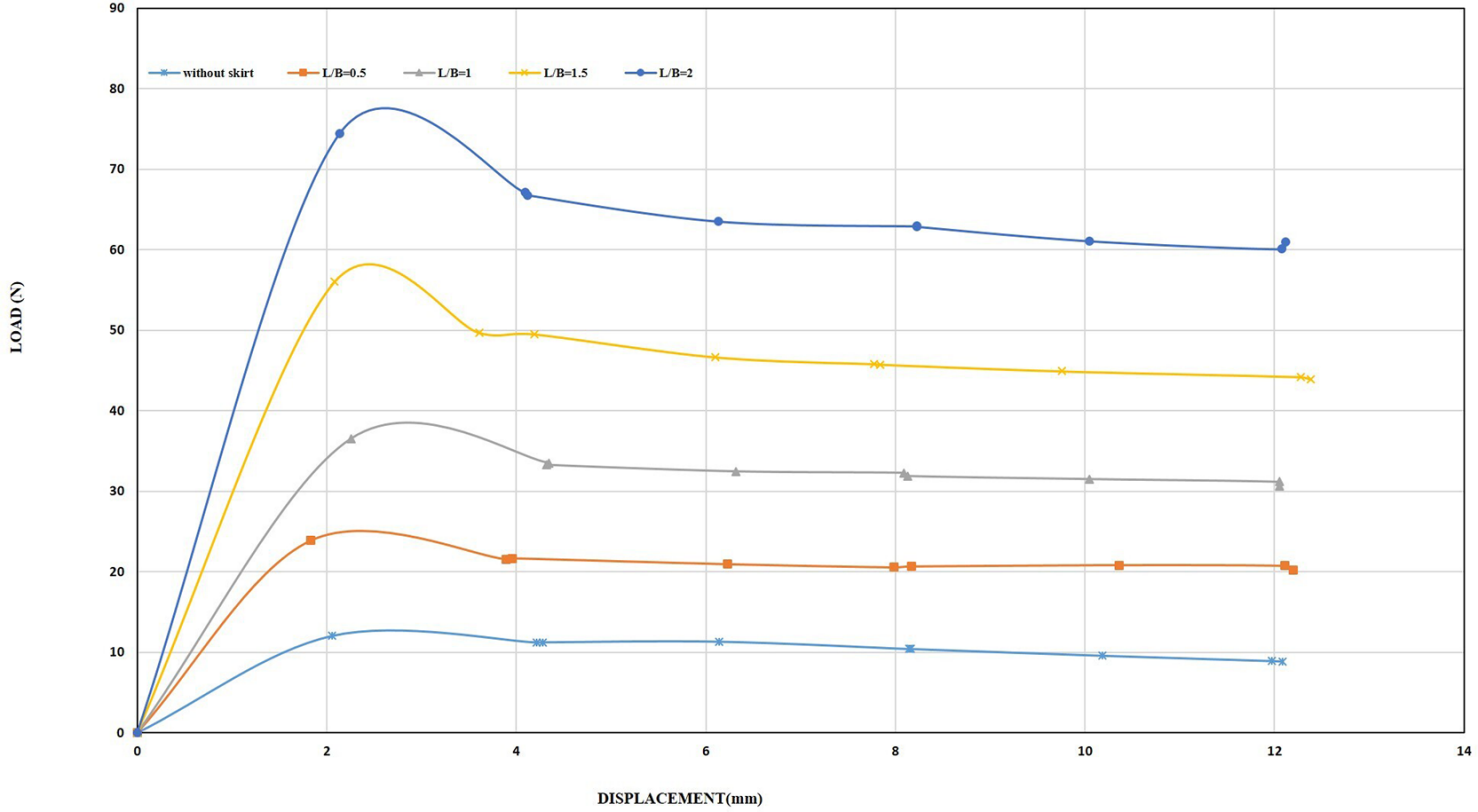
Load-displacement Relationships of Model Skirts of β =0°, and 2 mm/min Displacement Rate.

The uplift loading of the unmodified footing was 12.05 N and was considered as a reference for comparison with the uplift load obtained by adding a skirt to the footing. The uplift capacity of the shallow-skirted footing increased with increasing embedding ratio (L/B), as was the case with the first displacement rate, but it was lower than the value that obtained at the first displacement rate. Figure 29 showed the load-displacement relationships for standard skirted foundations.

The maximum uplift was observed to be 80.76 N for the L/B = 2, at 0.5 mm/min displacement rate, while it was 74.45 N for 2 mm/min, regardless of the embedding ratio. This behavior can be explained by the fact that the maximum uplift force is achieved at very small displacements and then gradually decreases with increasing displacement during the test, as showed in previous tests. However, the improvement rate increased by approximately 6.17 times compared to the unskirted footing, which is 5.81 times greater than the improvement rate for the first displacement rate. The above results are consistent with the result of Kulczykowski (2020). Tables 10 illustrated the results of all tests with various L/B and β.

**Table 10.** Uplift capacity values of skirted footings with 100 mm for different L/B ratios.

Skirt Length L (cm)	Uplift capacity(N)	Improvement %
0B	12.05	100
0.5B	23.87	198.09
1B	36.55	303.31
1.5B	56.00	464.77
2B	74.45	617.88

**4.2.2 The Effects of Inclination Angle of Skirt on uplift capacity at 2 mm/min Displacement Rate**

Increasing displacement ratio does not change the general behavior of the skirt's uplift resistance by increasing inclination angle compared to the behavior of the skirt by increasing embedment ratio. However, the difference lies in the values of the soil improvement ratio. The maximum load that the skirt can achieve occurs with a diminutive displacement, after which it gradually decreases until the residual load is reached. This behavior applies to the skirt with an inclination angle of 0° and for all embedment ratios, as well as for all inclination angles (15°, 30°, and 45°) and L/B of 0.5, and for both skirt types (chamfered and straight corners).

**Figure 30.**
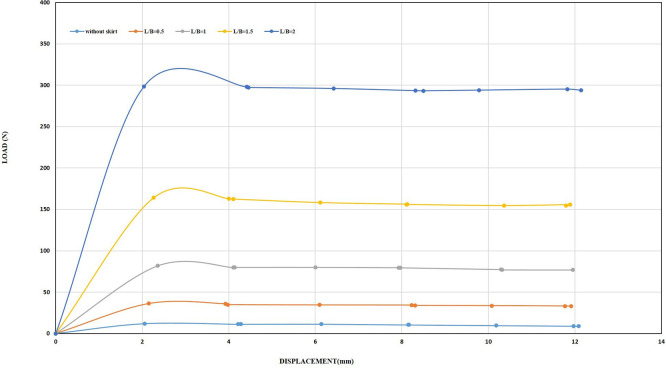
Load-Displacement Relationships of Model Skirts (Chamfered corners) at β =45° and 2 mm/min Displacement Rate.

**Figure 31.**
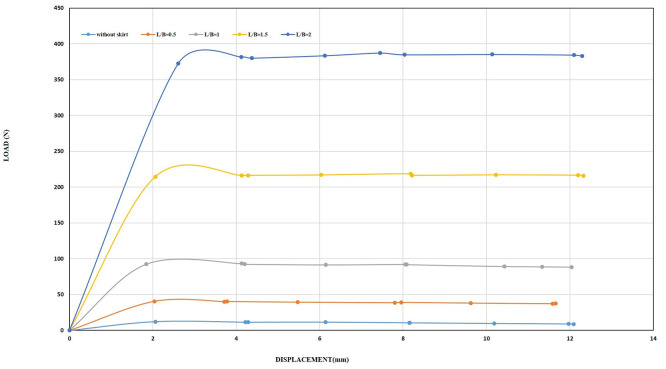
Load-Displacement Relationships of Model Skirts (Straight corners) at β =45° and 2 mm/min Displacement Rate.

For skirts with an inclination angles of (15°, 30°, and 45°), the peak uplift resistance was achieved at a diminutive displacement too, then decreases with slight displacement. Thereafter, the uplift resistance increases again until it reaches its maximum value at varying displacement, followed by a further decrease in uplift resistance. Figures 30– 31 showing the results obtained.

The maximum uplift loading was observed to be 33.66 N and 34.14 N for the chamfered and straight corners, respectively, at L/B of 0.5, inclination angle of β = 45°, and a displacement rate of 0.5 mm/min, which is 2.42 and 2.45 times greater than the uplift forces for the unskirted footing. Meanwhile, the maximum uplift forces were 36.20 N and 40.49 N for the chamfered and straight corners, respectively, at the same embedment ratio and inclination angle but with a displacement rate of 2 mm/min, which is 3 and 3.36 times greater than the uplift forces for the unskirted footing.

Note that the maximum uplift forces were 269.68 N and 360.47 N for the chamfered and straight corners, respectively, at L/B of 2, an inclination angle of β = 45°, and a displacement rate of 0.5 mm/min. This value is equivalent to 19.42 and 25.97 times greater than that of a skirted footing without a skirt. Meanwhile, the maximum uplift forces were 298.42 N and 381.94 N for the chamfered and straight corners, respectively, at the same embedment ratio and inclination angle, but with a displacement rate of 2 mm/min. This load is equivalent to 24.76 and 31.69 times greater than the uplift force of an unskirted footing.

These results demonstrate the potential benefit of using a skirt for shallow footings, as it provides greater uplift resistance when subjected to rapid uplift forces, which can be considered an additional safety factor during design. Results above also comply with the results obtained by ( Kulczykowski, 2020). Tables 11 and 12 illustrated the results of uplift capacity for various L/B and β.

**Table 11.** Uplift Capacity Values of Chamfered-corner Skirted Footings for Different L/B Ratios, β, B= 100 mm, and 2mm/min Displacement Rate.

Skirt Length L (cm)	Skirt Inclination angle (β)	Uplift capacity(N)	Improvement %
0B		12.05	100
0.5B	15°	30.79	255.58
	30°	67.35	558.95
	45°	121.06	1004.62
1B	15°	224.16	1860.22
	30°	33.00	273.88
	45°	76.82	637.51
1.5B	15°	154.03	1278.26
	30°	287.61	2386.76
	45°	36.20	300.47
2B	15°	81.88	679.51
	30°	164.02	1361.15
	45°	298.42	2476.44

**Table 12.** Uplift Capacity Values of Straight-Corners Skirted Footings for Different L/B Ratios, β, B= 100 mm,and 2mm/min Displacement Rate.

Skirt Length L (cm)	Skirt Inclination angle (β)	Uplift capacity(N)	Improvement %
0B		12.05	100
0.5B	15°	34.55	286.73
	30°	67.39	559.28
	45°	126.79	1052.15
1B	15°	177.04	1469.21
	30°	39.21573	325.4282
	45°	81.13696	673.3077
1.5B	15°	185.9765	1543.309
	30°	363.2662	3014.532
	45°	40.49087	336.0098
2B	15°	93.36624	774.7913
	30°	218.7392	1815.187
	45°	381.948	3169.561

**4.2.3 The Effects of Shape of Skirt on Uplift Capacity at 2 mm/min Displacement Rate**

The effect of shape on uplift capacity is greater for skirts with L/B more than 0.5 for both types (chamfered and straight corners). As for increasing the displacement rate, its effect did not differ from the parameters discussed previously. Here, a comparison can be made between the results obtained at a displacement rate of 0.5 mm/min and those obtained at a displacement rate of 2 mm/min.

The skirts with chamfered edges and straight angles with an embedding ratio of 0.5, inclination angle of 15°, and a displacement rate of 0.5 mm/min showed uplift resistance values of approximately 33.66 N and 34.14 N, respectively. With increasing displacement ratio, the uplift capacity of the straight-corner skirt increased by 40.49 N, which is greater than the value obtained from the chamfered skirt of 36.20 N. This behavior also applies to uplift values L/B of 2 and a 45° inclination angle.

The straight-corner skirts showed a significant improvement in resistance to uplift forces, increasing from 360.47 N for the first displacement ratio to 381.94 N for the second displacement ratio, compared to those with chamfered corners, which were approximately 269.68 N for the first displacement ratio and 298.42 N for the second displacement ratio. The uplift values for all L/B and inclination angles are listed in the Table 11 and 12. This is complies with the findings of ( Kulczykowski, 2020).

**4.2.4 The Effects of Adding Wings to the Skirt on Uplift Capacity at 2 mm/min Displacement Rate**

**Figure 32.**
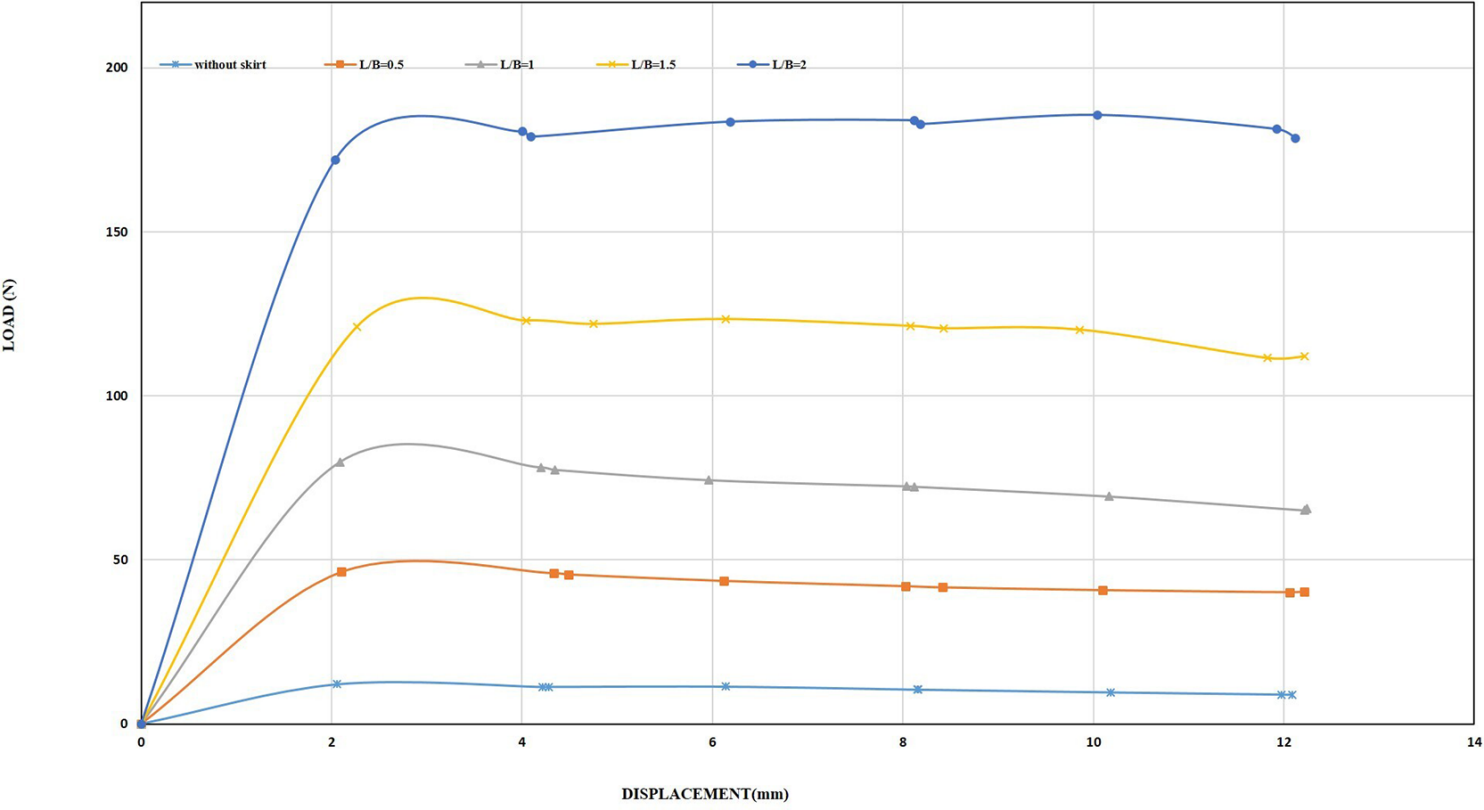
Load-Displacement Relationships of Model Skirts with Wings at β = 0° and 2 mm/min Displacement Rate.

The effect of increasing the displacement rate on the uplift force using the wings with the skirt was no different from its effect when using the other parameters discussed previously. The results show that the uplift resistance recorded 48.79 N for the skirt at 0° and L/B = 0.5 when applying a displacement rate of 0.5 mm/min. Whereas applying a displacement rate of 2 mm/min and the same parameters recorded 46.24 N, which is the same behavior as the skirt at 0°, as shown in Figure 32. and Table 13.

**Table 13.** Uplift Capacity Values of Skirted Footings for Different L/B Ratios, B=100 mm, β = 0°, and 2mm/min Displacement Rate with Wings.

Skirt Length L (cm)	Uplift capacity(N)	Improvement %
0B	12.05	100
0.5B	46.2468	383.7749
1B	79.86502	662.7526
1.5B	122.9932	1020.648
2B	180.6104	1498.779

**Figure 33.**
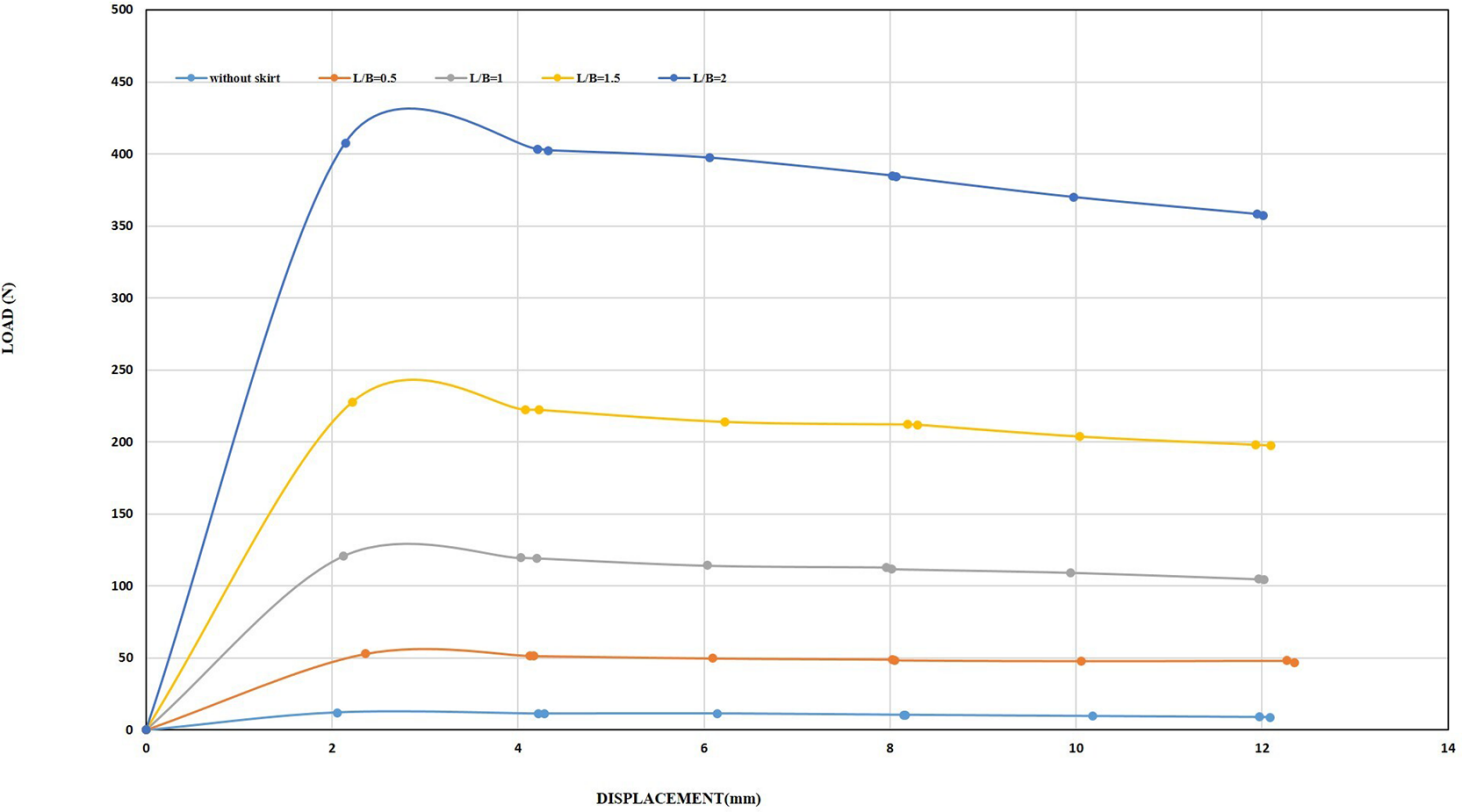
Load-Displacement Relationships of Model Skirts (Chamfered Corners) with Wings at β =45° and 2 mm/min Displacement Rate.

**Figure 34.**
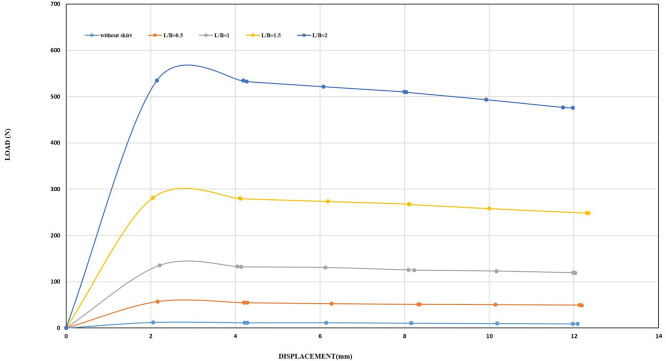
Load-Displacement Relationships of Model Skirts (Straight Corners) with Wings at β =45° and 2 mm/min Displacement Rate.

The skirt with L/B of 2, inclination angle of 45°, and a displacement rate of 0.5 mm/min had uplift load of 376 N for chamfered skirt and 525.59 N for the straight skirt, while applying a displacement rate of 2 mm/min recorded 407.53 N and 534.68 N for chamfered and straight skirts, respectively as shown in Figures 33 to 34.

Tables 14 and 15 shows all the results obtained from applying a displacement rate of 2 mm/min for all L/B and β value. From what was mentioned above, the skirts, both chamfered and straight-cornered, provided higher resistance to uplift load under rapid uplift loads, which can be considered a safety factor that can be added to the design calculations. This is comply with the findings of ( Kulczykowski, 2020). By comparing the results between the 0.5 and 2 mm/min, it was found that some of the uplift capacity values are close. This can be explained by the fact that changing the displacement rate from 0.5 to 2 mm/min is very small and equals 1.5 mm/min due to the limited capabilities of the apparatus. Therefore, the percentage improvement in uplift capacity is close.

**Table 14.** Uplift Capacity Values of Chamfered-Corners Skirted Footings for Different L/B Ratios, B= 100 mm, β, and 2mm/min Displacement Rate with Wings.

Skirt Length L (cm)	Skirt Inclination angle (β)	Uplift capacity(N)	Improvement %
0B		12.05	100
0.5B	15°	47.45	393.83
	30°	98.80	819.90
	45°	179.65	1490.88
1B	15°	289.33	2400.98
	30°	50.15	416.23
	45°	114.25	948.11
1.5B	15°	214.69	1781.64
	30°	373.17	3096.76
	45°	52.90	439.05
2B	15°	120.84	1002.81
	30°	227.70	1889.55
	45°	407.53	3381.91

**Table 15.** Uplift Capacity Values of Straight-Corners Skirted Footings for Different L/B Ratios, B= 100 mm, β, and 2mm/min Displacement Rate with Wings.

Skirt Length L (cm)	Skirt Inclination angle (β)	Uplift capacity(N)	Improvement %
0B		12.05	100
0.5B	15°	49.65	412.02
	30°	107.80	894.57
	45°	190.01	1576.79
1B	15°	329.84	2737.18
	30°	54.60	453.12
	45°	127.10	1054.78
1.5B	15°	256.81	2131.16
	30°	448.26	3719.91
	45°	57.10	473.87
2B	15°	135.23	1122.21
	30°	281.40	2335.24
	45°	534.68	4436.99

**4.2.5 Uplift Capacity of Single Pile under 2 mm/min Displacement Rate**

Although the displacement rate was increased to 2 mm/min, the results obtained from the test did not show a significant improvement in the uplift capacity of shallow footing compared to the results obtained from the skirted footing tests at the same displacement rate. The maximum uplift resistance was recorded at 52 N for the footing with a single closed-end pile at an embedding ratio of 15, while the maximum uplift resistance for the straight-cornered skirted footing at the same displacement rate, embedding ratio of 2, and an inclination angle of 0° was 74 N, which is 1.42 times greater than that of the pile footing.

However, the results obtained from the test of the straight-cornered winged skirted footing at an embedding ratio of 2 and an inclination angle of 45° equaled 534 N as the maximum uplift resistance, which is 10.25 times greater than the uplift capacity of the pile footing. It is worth noting here that the piles used under the footing were inserted under compression load for 8 cm into the soil sample to gain additional resistance, which contributes to the uplift capacity. However, the results obtained from the pile footing tests achieved lower uplift values compared to those obtained by using skirts.

**Figure 35.**
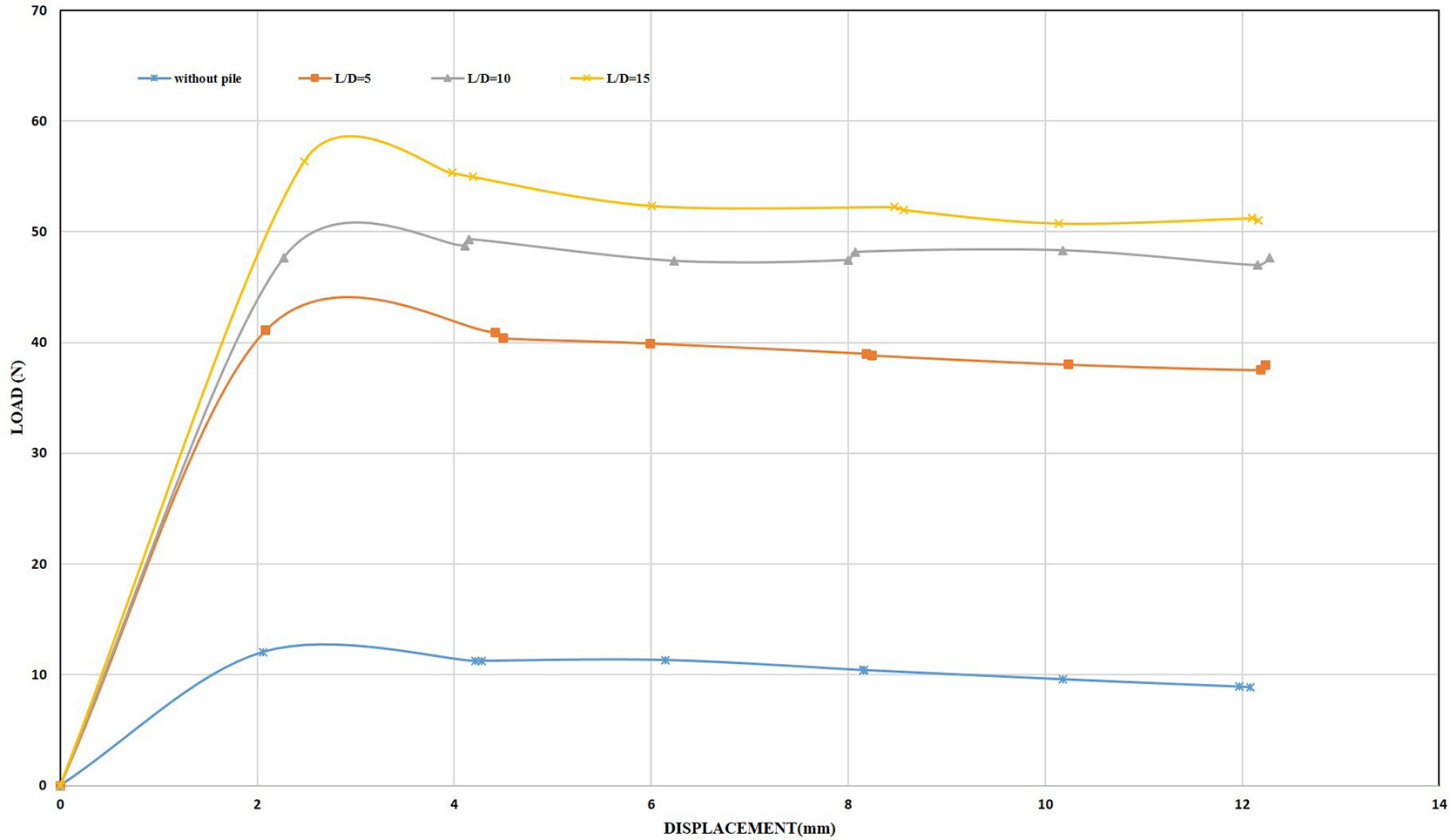
Load-Displacement Relationships of Single Closed End Pile at 2 mm/min Displacement Rate.

**Figure 36.**
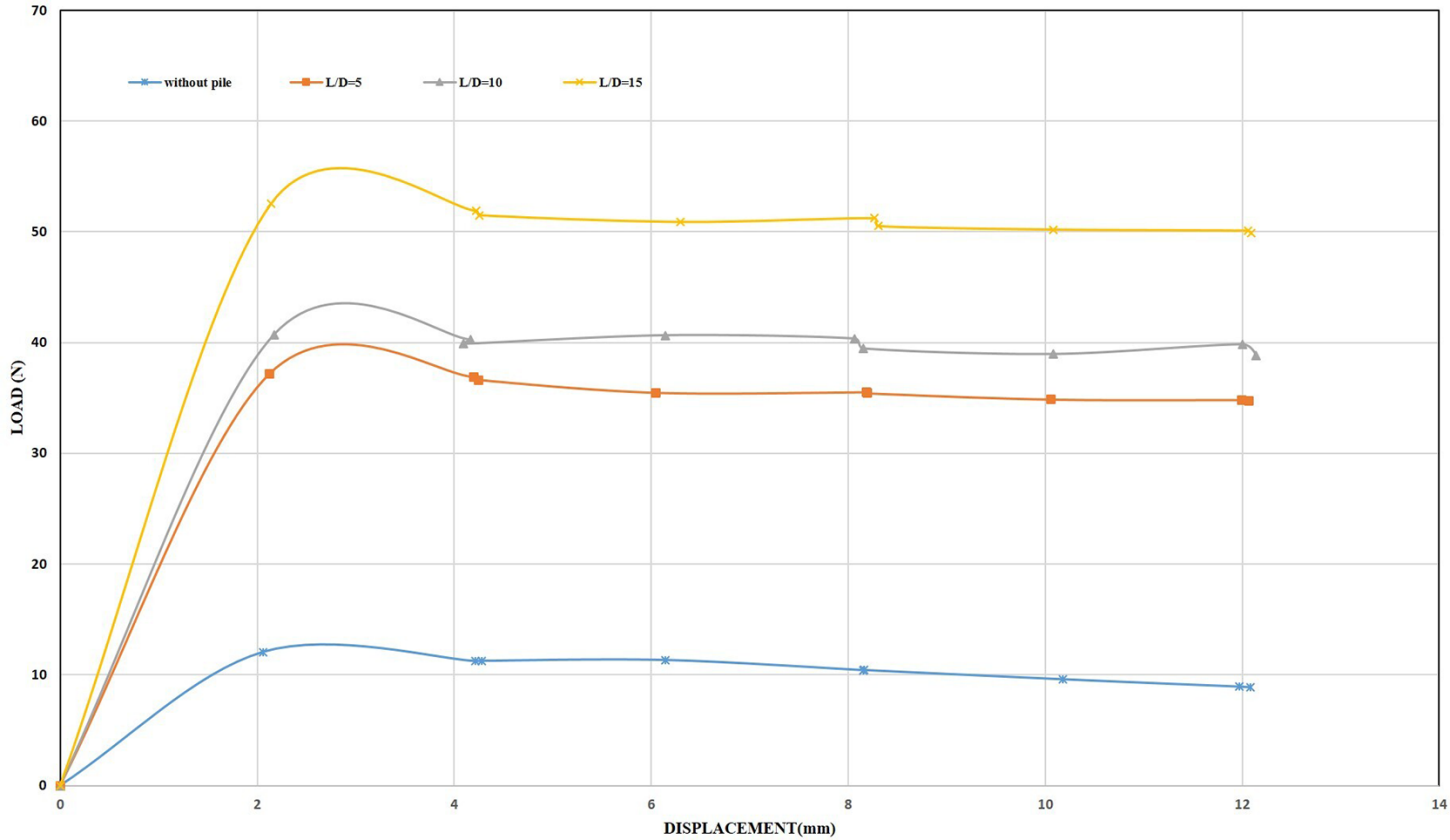
Load-Displacement Relationships of Single Opened End Pile at 2 mm/min Displacement Rate.

The behavior of the open-ended pile foundation did not differ significantly when the displacement rate increased compared to its behavior at a displacement rate of 0.5 mm/min. By analyzing the test results for both types of piles and comparing them with the results obtained from the skirt test, it was possible to prove that the skirts are a successful and economical alternative to deep foundations (piles). Figures 35 and 36 and Tables 16 and 17 show the results and uplift capacity values for the closed-ended and open-ended pile foundations.

**Table 16.** Uplift Capacity Values of Single Pile Footings for Different L/D Ratios of Closed End Piles, B=100 mm, and 2mm/min Displacement Rate.

Pile Length L (cm)	Uplift capacity(N)	Improvement %
Unskirted footing	12.05	100
5D	41.10	341.08
10D	49.33	409.43
15D	56.38	467.89

**Table 17.** Uplift Capacity Values of Single Pile Footings for Different L/D Ratios of Opened End Piles, B=100 mm, and 2mm/min Displacement Rate.

Pile Length L (cm)	Uplift capacity(N)	Improvement %	
Unskirted footing	0B	12.05	100
5D	5D	37.18	308.56
10D	10B	40.72	337.97
15D	15B	52.54	436.03

## Conclusions

Based on the results obtained from laboratory tests conducted on the shallow foundation under the influence of uplift loadings, the following conclusion was reached:

1. The uplift capacity of skirted footing increased with an increased L/B ratio of 2 at maximum value, which is about 6 times more than the un-skirted footing.
2. The uplift capacity of skirted footing increased with increased inclination angles at β = 45° and L/B ratio of 2 at maximum value, which is about 26 times more than the unmodified footing.
3. The geometric shape of the skirt substantially influences the uplift loads, as the maximum uplift force was obtained at the L/B ratio of 2, and β = 45° was 26 times for straight angle corners compared with 19 times for the chamfered corners, more than the unmodified footing.
4. The load-displacement behavior of the skirted footing on loose sand is nearly similar in all conditions in general trend but different in values and curve shapes. The ultimate pullout resistance of the skirt was attained for small values of L/B and β, but with high values of L/B and β, it reached the peak value.
5. The skirt length has less effect than the angle of skirt inclination.
6. The addition of wings to the lower edge of the skirt resulted in significant improvements in uplift capacity, acting as an anchor to secure the skirt to the soil.
7. The skirt footings are considered a successful alternative to the use of uplift piles in terms of safety.
8. The performance of the skirted footing and its behavior is largely consistent with the behavior of belled piles.
9. The best location of a bell is at the base of the pile, but for the skirted footing, the skirt connects to the footing at a shallow depth and extends to the specified depth.
10. By increasing the displacement rate, high values of uplifting resistance are achieved, and thus this increase in uplift capacity can be considered as an additional safety factor when designing.

## Data Availability

Zenodo: Laboratory Test Results for Sandy Soil Properties. https://doi.org/10.5281/zenodo.18205878 [ Al-zubaidi and Al- Saidi, 2026]. This project contains the following underlying data:
• Data Availability Statement.xlsx (Dataset containing results for Direct Shear Test, Sieve Analysis, Relative Density, Specific Gravity, and Raining Technique). Data are available under the terms of the Creative Commons Attribution 4.0 International license (CC-BY 4.0).

## References

[ref1] Abbas HO : The compressive capacity of conventional and under-reamed piles in soft clay. *IOP Conference Series: Materials Science and Engineering.* IOP Publishing;2021; Vol.1076(1): p.012094. 10.1088/1757-899X/1076/1/012094

[ref2] Acosta-Martinez HE Gourvenec SM Randolph MF : Effect of gapping on the transient and sustained uplift capacity of a skirted foundation in clay. *Soils Found.* 2010;50(5):725–735.

[ref3] Ahmed BA Saleh HM Jameel MM : Evaluation of Skirt-Raft Foundation Performance Adjacent to Unsupported Excavations. *Civil Engineering Journal (Iran).* 2024;10(12):4083–4103. 10.28991/CEJ-2024-010-12-018

[ref4] Jawad AS : Reliability analysis of the seismic stability of embankments reinforced with stone columns. *Journal of Engineering/College of Engineering/University of Baghdad.* 2011;17(04):2011. 10.31026/j.eng.2011.04.14

[ref5] Al-Mosawe MJ Al Saidi AA Jawad FW : Bearing capacity of square footing on geogrid reinforced loose sand to resist eccentric load. *Journal of Engineering/College of Engineering/University of Baghdad.* 2010;16(02):2010. 10.31026/j.eng.2010.02.17

[ref6] Al -Mosawe MJ Fattah MY Al-Zayadi AAO : Experimental Observations on the Behavior of A Piled Raft Foundation. *J. Eng. Des.* 2011;17(04). 10.31026/j.eng.2011.04.13

[ref7] Al-Mosawe MJ Al-Saidi A’a AH Jawad FW : Experimental and Numerical Analysis of Piled Raft Foundation with Different Length of Piles under Static Loads. *J. Eng. Des.* 2013;19(05). 10.31026/j.eng.2013.05.02

[ref8] Al Dabi SK Albusoda BS : Loosely Skirted Circular Foundation under Different Loading Conditions: Performance, Mechanism, and Limitations. *Eng Technol Appl Sci Res.* 2024;14(5):17464–17471. 10.48084/etasr.8421

[ref9] Al-Wakel SFA Mahmoud MR Abdulrasool AS : Experimental Studies and Finite Element Modeling of Piles and Pile Groups in Dry Sand under Harmonic Excitation. *J. Eng. Des.* 2014;20(07). 10.31026/j.eng.2014.07.04

[ref10] Andersen KH Murff JD Randolph MF : Suction anchors for deepwater applications. *Proceedings of the International Symposium on Frontiers in Offshore Geotechnics (ISFOG).* Perth, Western Asustrlia: CRC Press/Balkema;2005; pp.3–30.

[ref11] Al-Zubaidi AJ Al-Saidi A’a AH : Improving the Performance of Shallow Footing Subjected to Uplift Loading Using Structural Skirt. *Civ Eng J.* 2025;11(08). 10.28991/CEJ-2025-011-08-08

[ref12] Bachay HA Al-Saidi AAH : The optimum reinforcement layer number for soil under the ring footing subjected to inclined load. *Journal of Engineering/College of Engineering/University of Baghdad.* 2022;28(12). 10.31026/j.eng.2022.12.02

[ref13] Colliat JL Boisard P Gramet J-C : Design and installation of suction anchor piles at a soft clay site in the gulf of guinea. *Offshore Technology Conference.* 1996. 10.4043/8150-MS

[ref14] Daibil AR Al-Saidi AAH : The soil-anchors system theories and improvement: a review study. *Journal of Engineering/College of Engineering/University of Baghdad.* 2025;31(7):2025. 10.31026/j.eng.2025.07.10

[ref15] Dickin EA Leung CF : The influence of foundation geometry on the uplift behavior of piles with enlarged bases. *Can. Geotech. J.* 1992;29(3):498–505. 10.1139/t92-054

[ref16] Emirler B Tolun M Yildiz A : Investigation on determining uplift capacity and failure mechanism of the pile groups in sand. *Ocean Eng.* 2020;218. 10.1016/j.oceaneng.2020.108145

[ref17] Farokhi AS Alielahi H Mardani Z : Optimizing the performance of under-reamed piles in clay using numerical method. *Electron. J. Geotech. Eng.* 2014;19(Bundle G):1507–1520.

[ref18] Honda T Hirai Y Sato E : The uplift capacity of belled and multi-belled piles in dense sand. *Soils Found.* 2011;51(3):483–496. 10.3208/sandf.51.483

[ref19] Ilamparuthi K Dickin EA : The influence of soil reinforcement on the uplift behavior of belled piles embedded in the sand. *Geotext. Geomembr.* 2001;19(1):1–22. 10.1016/S0266-1144(00)00010-8

[ref20] Kelly RB Byrne BW Houlsby GT : Tensile loading of model caisson foundations for structures on sand. *Proceedings of the International Symposium of Offshore and Polar Engineering.* ISOPE;2004;638–641.

[ref21] Kulczykowski M : Experimental investigation of skirted foundation in sand subjected to rapid uplift. *Arch Hydroeng Environ Mech.* 2020;67(1–4):17–34.

[ref22] Kang JG Kang GO : Experimental and Semitheoretical Analyses of Uplift Capacity of Belled Pile in Sand. *Int J Geomech.* 2022;22(12):04022217. 10.1061/(ASCE)GM.1943-5622.0002511

[ref23] Landlin G Chezhiyan S : Behaviour of skirt foundation on loose sea sand with pullout loading under dry and submerged conditions. *International Journal of Civil Engineering and Technology (IJCIET).* 2017; Volume8(4): pp.1897–1904. April 2017. Article ID: IJCIET_08_04_216.

[ref24] Ahmadi M Ghazavi M : Effect of skirt geometry variation on uplift capacity of skirted foundation. *International Offshore and Polar Engineering Conference.* 2012; pp695–699.

[ref25] Merifield RS Sloan SW : The ultimate pullout capacity of anchors in frictional soils. *Can. Geotech. J.* 2006;43(8):852–868.

[ref26] Mohamed A Amr H : Contribution of vertical skin friction to the lateral resistance of large-diameter shafts. *J. Bridg. Eng.* 2014;19(2):289–302. 10.1061/(ASCE)BE.1943-5592.0000505

[ref27] Nariyelil AJ Cyrus S Abraham BM : Experimental Analysis of Uplift Behavior in Under-reamed Piles: A Comparative Study in Saturated Clay and Clayey Sand. *Indian Geotech. J.* 2025;56:1462–1477. 10.1007/s40098-025-01229-z

[ref28] Parthipan N Kumar M : Experimental Study on Uplift Load Carrying Capacity of Steel Pile in Sand. *International Journal of Science and Research (IJSR).* 2015;6(5):2682–2684.

[ref29] Salah Alhalbusi G Al-Saidi AAH : Enhancing the ability of the square footing to resist positive and negative eccentric-inclined loading using an inclined skirt. *E3S Web of Conferences.* 2023;427:01020. 10.1051/e3sconf/202342701020

[ref30] Salih SJH Salih NB Noory DB : Load-Settlement Behavior of Steel Piles in Different Sandy Soil Configurations. *J. Eng. Des.* 2020;26(10). 10.31026/j.eng.2020.10.08

[ref31] Chatterjee S Mana DSK Gourvenec S : Large deformation numerical modeling of the short-term compression and uplift capacity of offshore shallow foundations. *ASCE J. Geotechnical and Geoenvironmental Engineering.* 2014;140(3).

[ref32] Shakir ZH : Improvement of gypseous soil using cutback asphalt. *Journal of Engineering/College of Engineering/University of Baghdad.* 2017;23(10). 10.31026/j.eng.2017.10.04

[ref33] El-Gharbawy S Olson R : The cyclic pullout capacity of suction caisson foundations. *9th International Offshore and Polar Engineering Conference.* Brest, France:1998. May 1999.

[ref34] Singh RP Dubey CS Singh S : A new slope mass rating in mountainous terrain. Jammu and Kashmir Himalayas: applicationof geophysical technique in slope stability studies. *Landslides.* 2013;10(3). 10.1007/s10346-012-0323-y

[ref35] Stove OJ Bysveen S Christophersen HP : New foundation systems for the snorre development. *Proc. Annual Offshore Technology Conf.; Houston, Paper OTC 6882.* 1992.

[ref36] Tjelta TI Haaland G : *Novel foundation concept for a jacket finding its place.* Offshore Site Investigation and Foundation Behaviour; Soc. for Underwater Technology;1993; vol.28:717–728.

[ref37] Tomlinson Woodward : *Pile Design and Construction Practice.* London: CRC Press; 5th ed 2007. 10.4324/9780203964293

[ref38] Turner JR Kulhawy FH : *Experimental analysis of drilled foundations subjected to repeated axial loads under drained conditions. Report EL-S32S.* Palo Alto, California: Electric Power Research Institute;1987.

[ref39] Wang X Zeng X Li J : Vertical performance of suction bucket foundation for offshore wind turbines in sand. *Ocean Eng.* 2019;180:40–48. 10.1016/j.oceaneng.2019.03.049

[ref40] Watanabe T Hama K Horii Y : Study on Uplift Capacity of Belled Pile in Sandy Soil. Duc Long P Dung NT , editors. *Proceedings of the 5th International Conference on Geotechnics for Sustainable Infrastructure Development. GEOTEC 2023. Lecture Notes in Civil Engineering.* Singapore: Springer;2024; vol395. 10.1007/978-981-99-9722-0_7

[ref41] Li X Gaudin C Tian Y : Effect of perforations on uplift capacity of skirted foundations on clay. *Can. Geotech. J.* 2013;51:322–331.

[ref42] Xie L Mas S Lin T : The seepage and soil slug formation in suction caissons in sand using visual tests. *Appl. Sci.* 2020;10:566.

[ref43] Zdravkovic L Potts DM Jardine RJ : A parametric study of the pull-out capacity of bucket foundations in soft clay. *Geotechnique.* 2001;1: page55–67.

[ref44] Al-zubaidi AJ Al- Saidi AA : Laboratory Test Results for Sandy Soil Properties.[Data set]. *Zenodo.* 2026. 10.5281/zenodo.18205878

