# Supplementary material for: Modifying the capacity of shallow foundations to resist uplift forces using structural skirts as a promising alternative to using piles
Source: F1000Res. 2026 Apr 16;15:531. [Version 1] doi: 10.12688/f1000research.175996.1 (PMC13191320; doi:10.12688/f1000research.175996.1)
Supplement: Peer review history

# Reviewer report: Reviewer response for version 1

**DOI:** 10.5256/f1000research.194022.r484786

**Published:** 2026-05-20

**Authors:** Eliomar Gotardi Pessoa

**Affiliations:** 1 UFRJ-Universidade Federal do Rio de Janeiro-RJ, Rio de Janeiro, Brazil

**Manuscript**

 Al-Zubaidi & Al-Saidi, 2025/2026

**Journal**

 F1000Research

**Field**

 Geotechnical Engineering / Foundation Design

 1. Article Summary

 This experimental study investigates whether structural skirts attached to shallow footings can serve as a viable and more economical alternative to single piles for resisting uplift forces. The authors tested a 10 cm x 10 cm square footing model on loose sand (relative density 30%) using two groups of skirts — chamfered and straight corners — with varying embedment ratios (L/B = 0.5 to 2.0), inclination angles (0°, 15°, 30°, 45°), and the addition of lateral wings to the lower edges of the skirts. Two displacement rates (0.5 and 2 mm/min) were applied. Results were compared against single closed-end and open-end steel piles at embedment ratios of 5D, 10D, and 15D.

 The main finding is that skirted footings with L/B = 2, a 45° inclination angle, straight-corner geometry, and lateral wings achieved uplift resistance up to 9 times greater than the best single pile tested (at 0.5 mm/min) and up to 10.25 times greater at 2 mm/min. The authors conclude that structural skirts are a technically superior and economically viable alternative to uplift piles in loose sandy soils.

 2. Detailed Review

 2.1 Clarity of Presentation and Literature

 The paper addresses a relevant and practically important topic in geotechnical engineering. The abstract is clear, and the introduction provides a reasonable context for the research. The literature review covers a reasonable range of prior work on skirted foundations and uplift piles. However, several presentation issues require attention:

- The Tables 1–17 in the submitted version are incomplete — many cells are truncated or missing values (e.g., Tables 3, 4, 6, 7, 11, 12, 14, 15, 17). All tables must be provided in full with complete numerical data.
- The figures reference GIF animations (e.g., Figure 1 flowchart, Figures 13–36), but no figures could be inspected during review. The authors must ensure all figures are high-resolution and embedded correctly in the final submission.
- Units are inconsistently presented. Forces are reported in Newtons (N) throughout the body, but the basis for improvement ratios (e.g., '9 times', '10.25 times') should be clearly defined relative to a single reference value stated prominently.
- The literature cited is generally relevant and reasonably current, though some references (e.g., Stove et al., 1992; Tjelta and Haaland, 1993) are quite dated and included primarily for historical context. More recent numerical studies on skirted foundations (post-2020) could be better represented.
- Minor language and grammar issues are present throughout the text (e.g., 'perres' in phrasing, inconsistent tense use). A thorough English language edit is recommended before final acceptance.

2.2 Study Design and Technical Soundness

 The experimental design is broadly appropriate for the stated objectives. The comparison between skirted footings and single piles under controlled laboratory conditions on loose sand is logical. However, the following technical concerns must be addressed:

- The use of only one relative density (Dr = 30%) limits the generalizability of the findings. The authors should either acknowledge this explicitly as a study limitation or provide at least one additional density for comparison.
- The piles were pre-loaded under compression for 8 cm into the soil before uplift testing. This pre-compression modifies the surrounding sand state and likely enhances pile uplift performance in a way that would not occur in typical field conditions. This procedural choice needs clear justification, and its effect on comparative fairness must be discussed.
- Only two displacement rates (0.5 and 2 mm/min) were tested, and the authors acknowledge the device's limited range. The practical relevance of this range to real seismic or wind-induced uplift events should be discussed more explicitly.
- The skirt embedment ratios tested (L/B up to 2, i.e., 20 cm depth for a 10 cm footing) and pile embedment ratios (up to 15D, i.e., 45 cm length for a pile inserted only 8 cm) result in a significant disparity in absolute embedded length. This asymmetry makes direct comparison between skirt and pile performance potentially misleading and should be discussed critically.
- The mechanism producing the 'zigzag' load-displacement curve at 0.5 mm/min is attributed to coarse grain interference. While plausible, this explanation should be supported by additional evidence or references.

2.3 Reproducibility of Methods

 The paper provides a reasonable description of the experimental apparatus, soil preparation procedure (raining technique), and test matrix. However, several details necessary for replication are missing or unclear:

- The exact sand raining height used to achieve Dr = 30% is stated as 224 mm, but the calibration curve or relationship between fall height, flow rate, and relative density should be provided or referenced more precisely.
- The dimensions and wall thickness of the skirts are stated, but the exact steel grade and surface finish (roughness) of both the skirts and piles are not specified. These parameters are known to influence skin friction and thus uplift capacity.
- The method for measuring pile and footing displacement using LVDTs is mentioned, but no information is provided on calibration, accuracy, or drift of the measurement system.
- The number of repeat tests per configuration is not stated. With 126 total tests across the configurations described, it appears each condition was tested only once, which raises concerns about repeatability and random variability in results (especially given the acknowledged non-uniform grain distribution).
- No information is given on how the footing was connected to the loading arm during uplift — whether the connection allowed rotation or was rigid — which affects the load transfer mechanism.

2.4 Statistical Analysis

 The paper does not employ any formal statistical analysis. Results are compared by computing improvement ratios and presenting load-displacement curves. While this approach is common in geotechnical model studies, the following points should be addressed:

- Given the acknowledged variability in sand grain distribution between tests, at least a subset of key configurations should be replicated (a minimum of three replicates is standard) to establish data variability and confidence in the reported values.
- The improvement ratios reported (e.g., 'about 9 times', '10.25 times') are presented without any measure of uncertainty. At minimum, the authors should discuss potential sources of experimental error and their estimated magnitude.
- The claim that some uplift capacities are 'close' between the two displacement rates due to the 'small difference of 1.5 mm/min' is not a rigorous statistical conclusion. A more principled discussion of the rate effect is required.

2.5 Data Availability

 The authors have deposited raw data (Direct Shear Test, Sieve Analysis, Relative Density, Specific Gravity, and Raining Technique results) on Zenodo under a CC-BY 4.0 license. This is commendable and meets F1000Research open data requirements. However:

- The Zenodo dataset does not appear to include the primary uplift load-displacement data from the 126 tests. These data are the core scientific output of the study and must be made available for full reproducibility.
- If raw LVDT and load cell time-series data exist, their deposition is strongly encouraged.

2.6 Conclusions

 The conclusions are broadly consistent with the results, but several concerns arise:

- Conclusion 7 states that 'skirt footings are considered a successful alternative to the use of uplift piles in terms of safety.' This claim is based on a single pile type tested at limited embedment ratios and without a true like-for-like comparison in terms of embedded length or cost. The conclusion should be stated with appropriate caveats.
- The economic argument (that skirts are more economical than piles) is asserted repeatedly but never substantiated with cost data or analysis. Either a preliminary cost comparison should be included or this claim should be removed or qualified.
- Conclusions 8 and 9 about the analogy between skirted footings and belled piles are interesting but unsupported by a formal comparison. These would benefit from a referenced discussion or a dedicated comparative analysis.
- The study is conducted entirely at model scale under 1g conditions. The influence of stress level on sand behaviour is well-established in the literature. The absence of any discussion of scale effects or centrifuge validation is a notable omission that limits confidence in the applicability of the findings to full-scale design.

3. Required Revisions

 The following items are considered essential for the manuscript to meet the standards for indexing:

1. Provide complete tables with all numerical data (Tables 3, 4, 6, 7, 11, 12, 14, 15, 17 are incomplete).
2. Deposit the full uplift load-displacement dataset (all 126 tests) in the Zenodo repository and update the data availability statement.
3. Provide at minimum three replicate tests for key configurations to assess variability and support the reported improvement ratios.
4. Justify and discuss the pre-compression loading of piles before uplift testing and its impact on the pile-vs-skirt comparison.
5. Discuss the scale effects and the limitations of 1g laboratory model testing for drawing conclusions applicable to field-scale design.
6. Remove or substantiate the claims regarding economic advantage of skirts over piles with supporting data.
7. Perform a thorough English language and grammar revision.

4. Minor Recommendations

- Consider adding a clear schematic diagram comparing the geometry of a skirted footing with that of a belled pile to support the analogy discussed in Conclusions 8 and 9.
- The zigzag curve behavior attributed to coarse grains merits a brief sensitivity discussion or cross-reference to a study on the effect of grain size on load-displacement noise.
- Inclusion of failure mode photographs for a wider range of configurations would strengthen the qualitative interpretation of the load-displacement curves.

**Overall Recommendation**

**Major Revision Required**

 The study addresses a topic of genuine practical interest and the experimental approach is reasonable in concept. However, incomplete data tables, absence of replicate tests, unsubstantiated economic claims, lack of scale-effect discussion, and concerns over the fairness of the pile-vs-skirt comparison must all be resolved before the work can be indexed. The authors are encouraged to resubmit after addressing the required revisions listed in Section 4.

Is the work clearly and accurately presented and does it cite the current literature?

Partly

If applicable, is the statistical analysis and its interpretation appropriate?

Partly

Are all the source data underlying the results available to ensure full reproducibility?

Yes

Is the study design appropriate and is the work technically sound?

Partly

Are the conclusions drawn adequately supported by the results?

Partly

Are sufficient details of methods and analysis provided to allow replication by others?

Partly

Reviewer Expertise:

Engineer and Master in Geotechnics

I confirm that I have read this submission and believe that I have an appropriate level of expertise to confirm that it is of an acceptable scientific standard.

# Reviewer report: Reviewer response for version 1

**DOI:** 10.5256/f1000research.194022.r479416

**Published:** 2026-05-15

**Authors:** K. V. S. B. Raju Raju, Dr. Lakshmi V

**Affiliations:** 1 University Visvesvaraya College of Engineering, Bangalore, Karnataka, India; 2 Civil Engineering, University of Visvesvaraya College of Engineering, Bengaluru, Karnataka, India

Please find the comments after reviewing the paper titled **“Modifying the capacity of shallow foundations to resist uplift forces using structural skirts as a promising alternative to using piles”.** Authors have made a good attempt to study the uplift capacity of skirted foundation experimentally and in addition also evaluated the effect of L/B ratio, inclination angle and wings.

 The research paper submitted by the authors needs a thorough review since it lacks clarity and basic understanding. The problem attempted is good but most of the parametric study in the needs proper technical explanation with proper citations.

 The major issues found with the studies and the writing are as follows.

1. The writing format, language/style of the manuscript needs to be checked (like spacing between words, grammar, spelling etc) (Ex: Pg No: 3, badly graded sand to be written as poorly graded sand.
2. How can the skirted foundations of different configuration be constructed in the field?
3. The relation between relative density and height of fall needs proper explanation. Was it possible to achieve relative density as low as 30%? Why the sand relative density of 30% is chosen. It’s not acceptable in general field conditions.
4. The sample preparation technique needs to be mentioned and how it is possible to achieve desired unit weight.
5. On what basis the different parameters like L/D ration, inclination angles are chosen and what is their practical applicability and justify.
6. In general, skirted foundations are used for offshore structures wherein the effect of water table plays a major role. Why authors have not made an attempt to study the effect of water table in enhancing uplift capacity.
7. What is the criteria adopted in determining the ultimate uplift capacity?
8. The uplift capacity was found to increase with L/B ratio and authors are suggested to explain about optimum L/B ratio wherein maximum capacity can be achieved.
9. Results of uplift capacities for various type of foundations could have presented graphically by considering different variables than presenting only in form of load-displacement graph.
10. How were the size effects and boundary effects due to rigid boundaries in your experimental work considered and minimized? Its seems to be completely violated in the present study.
11. It is suggested to provide a schematic sketch of the test set up

Is the work clearly and accurately presented and does it cite the current literature?

Yes

If applicable, is the statistical analysis and its interpretation appropriate?

Yes

Are all the source data underlying the results available to ensure full reproducibility?

Partly

Is the study design appropriate and is the work technically sound?

Yes

Are the conclusions drawn adequately supported by the results?

Yes

Are sufficient details of methods and analysis provided to allow replication by others?

Yes

Reviewer Expertise:

Strength and Dilatancy, Uplift, Lateral and oblique pullout capacity of various shapes of pile foundations like Belled Piles, Tapered Piles, Finned Piles, Step Tapered Piles and Helical Piles

We confirm that we have read this submission and believe that we have an appropriate level of expertise to confirm that it is of an acceptable scientific standard.
